# Necroptosis-related lncRNAs: Combination of bulk and single-cell sequencing reveals immune landscape alteration and a novel prognosis stratification approach in lung adenocarcinoma

**DOI:** 10.3389/fonc.2022.1010976

**Published:** 2022-10-20

**Authors:** Yizhu Yao, Liudan Gu, Ziyi Zuo, Dandan Wang, Tianlin Zhou, Xiaomei Xu, Lehe Yang, Xiaoying Huang, Liangxing Wang

**Affiliations:** The First Affiliated Hospital, Wenzhou Medical University, Wenzhou, China

**Keywords:** necroptosis, lung adenocarcinoma, neutrophil, immune infiltration, prognosis, single-cell sequencing, long non-coding RNA

## Abstract

Necroptosis, which is recently recognized as a form of programmed cell death, plays a critical role in cancer biology, including tumorigenesis and cancer immunology. It was recognized not only to defend against tumor progression by suppressing adaptive immune responses but also to promote tumorigenesis and cancer metastasis after recruiting inflammatory responses. Thus the crucial role of necrosis in tumorigenesis has attracted increasing attention. Due to the heterogeneity of the tumor immune microenvironment (TIME) in lung adenocarcinoma (LUAD), the prognosis and the response to immunotherapy vary distinctly across patients, underscoring the need for a stratification algorithm for clinical practice. Although previous studies have formulated the crucial role of lncRNAs in tumorigenicity, the relationship between necroptosis-related lncRNAs, TIME, and the prognosis of patients with LUAD was still elusive. In the current study, a robust and novel prognostic stratification model based on Necroptosis-related LncRNA Risk Scoring (NecroLRS) and clinicopathological parameters was constructed and systemically validated in both internal and external validation cohorts. The expression profile of four key lncRNAs was further validated by qRT-PCR in 4 human LUAD cell lines. And a novel immune landscape alteration was observed between NecroLRS-High and -Low patients. To further elucidate the mechanism of necroptosis in the prognosis of LUAD from a single-cell perspective, a novel stratification algorithm based on K-means clustering was introduced to extract both malignant and NecroLRS-High subsets from epithelial cells. And the necroptosis-related immune infiltration landscape and developmental trajectory were investigated respectively. Critically, NecroLRS was found to be positively correlated with neutrophil enrichment, inflammatory immune response, and malignant phenotypes of LUAD. In addition, novel ligand-receptor pairs between NecroLRS-High cells and other immunocytes were investigated and optimal therapeutic compounds were screened to provide potential targets for future studies. Taken together, our findings reveal emerging mechanisms of necroptosis-induced immune microenvironment alteration on the deteriorative prognosis and may contribute to improved prognosis and individualized precision therapy for patients with LUAD.

## 1 Introduction

With an estimated 1.8 million deaths, lung cancer is considered the leading cause of cancer deaths worldwide (1). Moreover, as the most common pathological subtype, lung adenocarcinoma (LUAD), contributes to a huge health care burden (2). Despite the growth of molecular targeted therapies and immune checkpoint inhibitors (ICIs) partly improving the overall survival (OS) of patients with LUAD, the prognosis is poor with a 5-year OS of only 21% (3). Due to the high tumor heterogeneity, it is still very hard to accurately predict the prognosis of patients individually. Thus, emerging approaches for predicting the prognosis and therapeutic effects in patients with LUAD are of crucial importance.

Apoptotic evasion and resistance trigger tumorigenesis and drug resistance, which result in chemotherapy failure. Hence, excluding strategies to overcome resistance to apoptosis, inducing non-apoptotic forms of programmed cell death is a promising option. Necroptosis, mainly mediated by Receptor-Interacting Protein Kinase 1 (RIPK1), Receptor-Interacting Protein Kinase 3 (RIPK3), and Mixed Lineage Kinase Domain-Like (MLKL) are being proposed as a novel programmed form of necrotic cell death (4). As an alternative form of regulated cell death, necroptosis mimics apoptosis as well as necrosis, with a highly controversial role in tumorigenesis. Crucial mediators of necroptosis pathway stimulate tumor metastasis and tumor progression (5–7), whereas evidence suggests that necroptosis plays an ‘immuno-sensitizer’ role, enhancing anti-tumor immunity by inducing and activating CD8^+^ T cells (8, 9). Given its critical role in cancer biology and tumor immune microenvironment, necroptosis is emerging as a novel target in cancer therapy, especially immunotherapy.

Immunotherapy was considered a great breakthrough and hope for patients with no added benefit from traditional treatment. Evidence suggested that the immune phenotypes defined by components of the tumor microenvironment (TME) affect patient prognosis and response to immunotherapy simultaneously (10). Jérôme Galon defined hot and cold tumors and creatively proposed a strategy to convert an immune cold into a hot tumor (11). However, the innate low sensitivity and drug resistance of immunotherapy hindered further treatment. Further elucidation of the mechanisms of immunotherapy resistance revealed insufficient immunogenicity resulting in an immunotherapy failure (12). Prior studies clarified that programmed cell death induced immunogenicity, and thus enhanced cancer immunotherapy (13). Consequently, necroptosis occurring in TME, especially involving immune cells, requires further exploration.

Long non-coding RNAs (lncRNAs) are being re-recognized and researched as emerging regulatory molecules that play a complex and precise regulatory role in the development and progression of cancer. The lncRNAs play a pivotal role in necroptosis *via* endogenous competition with miRNA and other signals (14–17). Additionally, the role of lncRNA in the tumor immune microenvironment has received considerable attention. A high level of lncRNA NKILA leads to elevated T cell sensitivity to activation-induced cell death (AICD) by inhibiting the NF-κB activity, which is associated with shorter patient survival (18). LINC00301 promoted Treg and repressed CD8 T cell infiltration by regulating the HIF1α pathway, which resulted in an immune-suppressing microenvironment (19). Hence, it is critical to identify necroptosis-related lncRNAs and acquire a deeper understanding of the interaction with immunotherapy.

**Figure 1:**
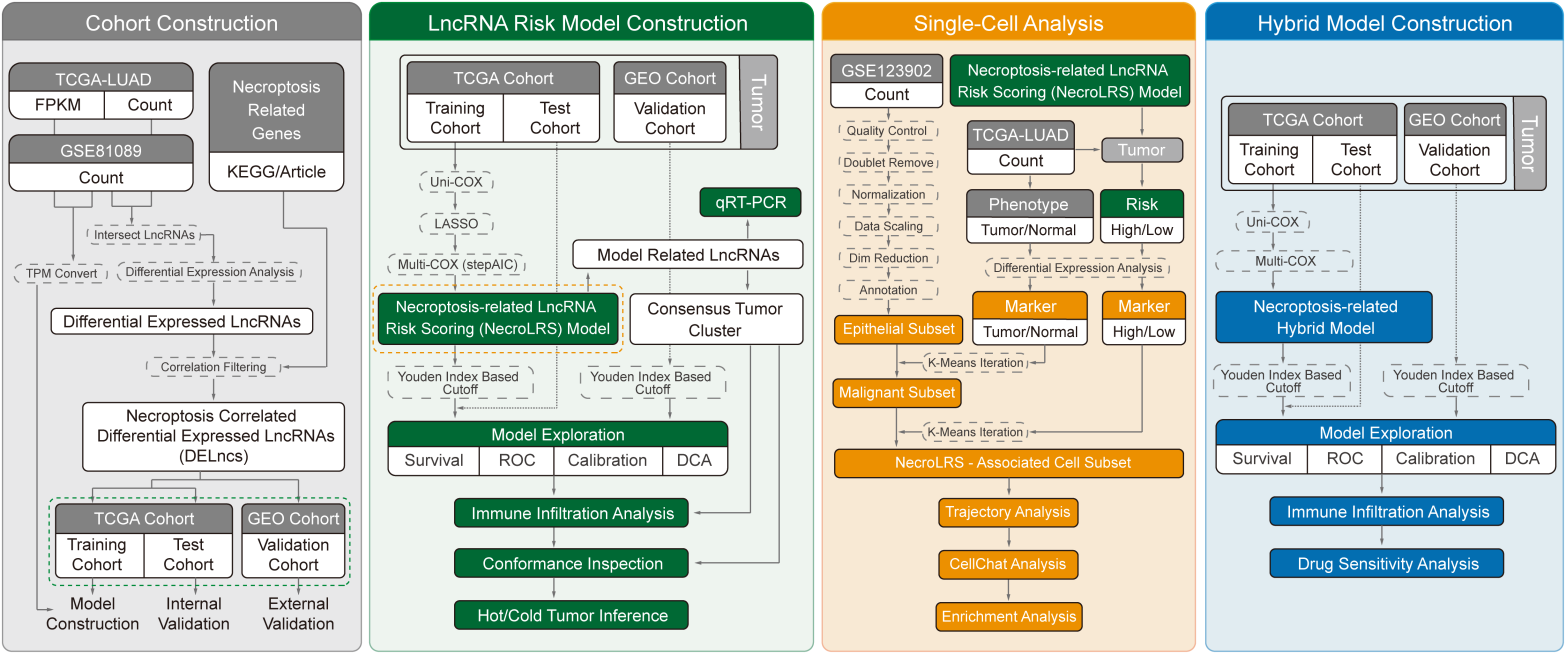
Flow diagram of the current study.

In this study, a robust and novel prognostic stratification model based on NecroLRS and clinicopathological parameters was constructed and validated. By combining both bulk- and scRNA-seq, we investigated the developmental trajectory transition of NecroLRS-related cell subtypes for the first time, and further explored the correlation between NecroLRS and tumor immune microenvironment. In addition, novel ligand-receptor pairs and optimal therapeutic compounds were investigated to provide potential targets for future studies. The detailed workflow of our study was clearly demonstrated in **Figure 1**. Taken together, our findings may contribute to improved prognosis and individualized precision therapy for patients with LUAD.

## 2 Materials and methods

### 2.1 Acquisition of necroptosis-related genes

The necroptosis-related genes were obtained from the Kyoto Encyclopedia of Genes, Genomes (KEGG) database (https://www.kegg.jp/) and the Molecular Signatures Database (MSigDB), combined with the necroptosis-related genes summarized by Zhao et al. (20). A total of 145 necroptosis-related genes were identified for further analysis (**Supplementary Table 1**).

### 2.2 Acquisition of transcriptome data

The RNA transcriptome profiling data (HTSeq-Counts and HTSeq-FPKM) were downloaded from The Cancer Genome Atlas (TCGA) through the R package “TCGAbiolinks” (21). Foremost, according to the human gene set annotation file of GRCh 38.105 version, we re-annotated the RNA transcriptome datasets from TCGA, followed by further distinguishing of lncRNAs and mRNAs. After filtering the duplicate samples, a total of 513 samples were recognized as tumor tissues and 59 samples as normal tissues. To reduce statistical bias, lung adenocarcinoma patients with missing overall survival (OS) values or unknown survival status were excluded, resulting in a final collection of 558 samples (Tumor patients, n = 499; Normal patients, n = 59). We subsequently transformed the transcript per million mapped reads (FPKM) data into transcripts per million (TPM) data. The count data were used only for Pearson correlation and differential expression analysis, while the TPM data were used for other downstream analyses. TCGA expression cohort was randomized into training (n = 313) and test (n = 186) cohort at a ratio of 6:4, while the expression profile of a GEO dataset (GSE81089) was acquired from GEO database (https://www.ncbi.nlm.nih.gov/geo/query/acc.cgi) and utilized as an external validation (n = 108), the baseline of the corresponding clinicopathological characteristics was demonstrated in **Supplementary Table 2**.

### 2.3 Acquisition of necroptosis correlated differential expressed lncRNAs

Co-expressed lncRNAs between TCGA and GSE81089 were first acquired, followed by differential expression analysis of tumor and normal tissues. Those with absolute log2 fold change (|log2FC|) > 1 and adjusted P-value < 0.05 were selected for the downstream analysis. A correlation analysis between necroptosis-related genes and differentially expressed lncRNAs was then performed, and those with absolute Pearson correlation coefficient >0.4 and P-value <0.001 were finally recognized as necroptosis-correlated differentially expressed LncRNAs (DELncs).

### 2.4 Construction and validation of NecroLRS and hybrid models

Univariate Cox regression was first used to determine prognosis-related necroptosis-correlated DELncs (p < 0.05). To avoid overfitting and resolve severe covariance, 10-fold cross-validated LASSO regression was performed. The iteration cycle was set to 1000, and prognosis-related necroptosis-correlated DELncs were further screened and filtered. Finally, the NecroLRS model was constructed based on multivariate step-Cox proportional risk analysis, and the risk score (NecroLRS) of each patient was calculated based on the coefficients of the corresponding variables. To reduce the heterogeneity between cohorts, the Youden index-based cut-off values were calculated independently in both TCGA and GEO cohorts and utilized to categorize patients into NecroLRS-High and -Low groups.

Multiple clinicopathological factors including NecroLRS, age, microsatellite instability score (MSIsensor), gender, race, AJCC stage, AJCC T, and AJCC N were utilized for hybrid model construction. AJCC M was excluded because of the large number of missing and uncertain values. Patients lacking complete clinical information (including missing values and uncertain stage definition) were deleted from the corresponding cohort. Clinicopathological factors of statistical significance (P-value < 0.05) in univariate Cox analysis were subjected to multivariate Cox analysis, and the variables with statistical significance were finally utilized to construct the hybrid model. Similarly, the risk score of the hybrid model was calculated as described before, while the cut-off values were obtained as described before. Additionally, all independent prognostic factors identified in the hybrid model were incorporated into the nomogram based on R package “rms”, in which the probability of 1-, 3-, and 5-year overall survival was further calculated.

In addition, Kaplan-Meier survival curves as well as receiver operating curves were obtained to determine the utility of NecroLRS and Hybrid models. Furthermore, calibration analysis was used to evaluate the accuracy of the model for predicting the overall survival of patients. Decision curve analysis (DCA) was used to explore the clinical utility of models in different cohorts.

### 2.5 Unsupervised clustering based on NecroLRS-related lncRNAs

Based on the expression profile of risk LncRNAs in the model as previously constructed, we performed unsupervised clustering using the R package “ConsensusClusterPlus” to explore the potential phenotype subgroups (22). Following dimension reduction *via* tSNE and PCA algorithms, the sample distribution was visualized using R packages “FactoMineR”, “factoextra”, and “Rstne”. The conformance between unsupervised clusters and NecroLRS-related patient groups was inspected based on Cohen’s Kappa value and visualized using a confusion matrix. Furthermore, the ESTIMATE and the ImmuneCellAI algorithms were used to determine immune infiltration in different subtypes (23, 24).

### 2.6 Exploration of the tumor immune landscape in bulk-seq

To explore the distribution of immune cells in NecroLRS-High and NecroLRS-Low groups, the abundance of immune cells was imputed using the ImmuneCellAI algorithm. Based on the expression profile, we quantitatively imputed the immune infiltration condition including the proportion of both immune and stromal cells of each sample based on the ESTIMATE algorithm. To elucidate the potential association between immune cell infiltration and NecroLRS, based on the expression profile of each immune cell, subgroups were created through the R package “survminer” and utilized for downstream analyses.

### 2.7 Prediction of drug sensitivity in bulk-seq

Based on the “oncopredict” R package” the drug sensitivity of each patient was imputed (25). The batch effects of expression matrix and CTRPv2 training matrix were first removed through the “Combat” algorithm (26–28), and the drug sensitivity (IC_50_) of each patient was inferred based on the CTRPv2 training matrix *via* ridge regression. The t-test was utilized to compare differences in IC_50_ between the different groups.

### 2.8 Functional enrichment and gene set enrichment analysis in bulk-seq

To validate the biological rationale of the NecroLRS model, risk lncRNA-related genes in the original necroptosis-related gene list were back-traced, then GO and KEGG pathway analyses were performed based on the R package “clusterProfiler” (29) and “org.Hs.eg.db”. In addition, GSEA 4.0.1 software was utilized for gene set enrichment analysis (GSEA) to assess trends in expression distribution across NecroLRS-High and -Low groups in the context of “c2.cp.kegg.v7.5.symbols.gmt” as a reference gene set. Pathways enriched in different groups were visualized using R package “ggplot2”, “grid”, and “gridExtra”.

### 2.9 qRT-PCR verification

Human bronchial epithelial cells (BEAS-2B) and human LUAD cell lines (HCC827, A549, PC9, and NCI-H1975) were purchased from the American Type Culture Collection (ATCC, United States). They were cultured in RPMI-1640 medium (Gibco, China) or high glucose Dulbecco’s Modified Eagle Medium (DMEM; Hyclone, Logan, UT, United States) supplemented with 10% fetal bovine serum (Gibco, China) at 37°C in an atmosphere of 5% CO_2_. Total RNA was extracted using the UNlQ-10 Column TRIzol Total RNA Isolation Kit (Sangon biotech) and reverse-transcribed with the TOROIVD^®^qRT Master Mix. qRT-PCR was performed using the Taq Pro Universal SYBR qPCR Master Mix (Vazyme). All samples were tested in triplicate. Primers were purchased from Generay Biotech Co., Ltd. (Shanghai, China) and listed in **Supplementary Table 3**. The differences in the expression of NecroLRS-related lncRNAs in each cell line were calculated *via* Mann-Whitney U tests.

### 2.10 Data pre-processing of single-cell RNA sequencing

A single-cell RNA sequencing (scRNA-seq) dataset (GSE123902) of patients with LUAD was acquired from the GEO database. After removing one sample after adjuvant therapy and another sample histologically diagnosed as large-cell lung carcinoma, a total of 6 samples acquired from 6 individuals were included in the scRNA-seq workflow. We performed a standard merge operation of these samples and the cells that satisfied the following criteria were included in downstream analysis: a) UMI counts > 1000; b) gene features more than 500 and less than 6000; c) percentage of mitochondria < 50%; and d) percentage of hemoglobin < 1%. After quality control, doublet estimation and removal were performed for each sample to calibrate the potential double-cell phenomenon, which might be introduced during the sequencing process. During the doublet removal, a standard Seurat process was first performed for each sample (including normalization, scaling, detection of highly variant genes, dimension reduction, and clustering). We assumed that each sample has a doublet cell rate (i represents each sample). A “doubletFinder_v3” function of R package “DoubletFinder” was used for each sample (30). We finally retrieved the singlet cells and merged them again into the working Seurat object (31).


$${\text{Doublet Rate}}_{i}={\text{Total Feature number}}_{i}\times 8\times 10^{-6}$$

After normalizing, data scaling, and PCA dimension reduction, cell clusters were acquired using the “FindNeighbors” and “FindClusters” functions based on the top 20 PCs of the PCA, and a resolution of 0.7, and the “tSNE” and “UMAP” algorithms were used for further dimension reduction and visualization of cell distribution. Based on the expression of cell markers reported previously (32), we first clustered cells into “Epithelial”, “Endothelial”, “Fibroblast”, “T”, “B”, “Mast”, and “Myeloid” categories. The “T” and “Myeloid” cells were separated for further dimension reduction and reclustering. “Myeloid” cells were further subdivided into “Macrophage”, “Neutrophil”, “Dendritic cells”, and “Monocyte”, while “T” cells were subdivided into “CD4^+^ T”, “CD8^+^ T”, “NK-T” cells. Additionally, “CD4^+^ T” cells were subdivided into “Treg” (regulatory T cells), “Tex” (Exhausted T cells), and “NOS” (Non-specific) cells, while “CD8^+^ T” cells were subdivided into “Tex” and “NOS” cells. All the cell clusters were visualized through “tSNE”. The expression of corresponding marker genes was visualized *via* violin and dot plots.

### 2.11 Detection of malignant and NecroLRS-related cell subsets

To facilitate the segregation of malignant cells from epithelial cells, Zhang et al. introduced a K-means cluster algorithm based on the expression features of cells (33). The same algorithm was used in the current study. First, TCGA-LUAD expression matrices (HTSeq-Count) were acquired and a standard limma differential expression analysis workflow was performed to obtain the top 50 significant gene signatures of both normal (“Non-malignant Marker_i_”) and tumor (“Malignant Marker_i_”) samples. The “Malignant Score” (*Sm*) and “Non-malignant Score” (*Sn*) of each cell were acquired using the “AddModuleScore” based on acquired markers. All analyzed features were binned into 25 bins, and the control features selected from the same bin were set to 100 per analyzed feature.

After acquiring the “Malignant” and “Non-malignant” scores, K-means clustering was performed to “initially” classify cells into “Malignant” and “Non-malignant” cells. During this process, the total number of centroids was 2. The mean *Sm* of cells from *a* = 1 …  *N* , where *N* is the number of cells whose centroid (*k*) = 1, and the mean *Sn* of cells from *b* = 1 …  *N*' , where *N*' is the number of cells whose *k* = 2 were obtained. Finally, the malignant centroid (*C*_*malignant*_ ) was identified based on the following criteria.


$$C_{malignat}=\{\begin{array}{c} 1if\left[Mean\left(\sum_{a=1}^{N}Sm_{a,k=1}\right)>Mean\left(\sum_{b=1}^{N'}Sm_{b,k=2}\right)\right]\wedge \left[Mean\left(\sum_{a=1}^{N}Sn_{a,k=1}\right)<Mean\left(\sum_{b=1}^{N'}Sn_{b,k=2}\right)\right]\\ 2if\left[Mean\left(\sum_{a=1}^{N}Sm_{a,k=1}\right)<Mean\left(\sum_{b=1}^{N'}Sm_{b,k=2}\right)\right]\wedge \left[Mean\left(\sum_{a=1}^{N}Sn_{a,k=1}\right)>Mean\left(\sum_{b=1}^{N'}Sn_{b,k=2}\right)\right] \end{array}$$

After initial clustering based on “Non-malignant Marker_i_” and “Malignant Marker_i_”, “FindMarkers” was used to identify the refined cell markers (“Non-malignant Marker_j_” and “Malignant Marker_j_”) across these two groups. The two markers were used in the new round of re-scoring and re-clustering based on the above-mentioned algorithm. After several iterations, the cell markers across two groups reached convergence. The final classification of cell type was used in the downstream analysis. The same workflow was also utilized to detect the NecroLRS-related cell subsets based on the differential gene signatures expressed in NecroLRS-High and -Low groups in bulk-seq analysis. Finally, the density and distribution plots were further performed to visualize the results of classification.

### 2.12 Biofunction prediction in scRNA-seq

To identify the pathways and GO terms enriched in the NecroLRS-High cell subset, GO and KEGG analyses were performed based on the up-regulated features in NecroLRS-High compared to the NecroLRS-Low cell subset. In the GO analysis, the top 10 terms that corresponded to each sub-ontology were identified using the dot plot, while in the KEGG analysis, the top 30 pathways were demonstrated. In addition, the “Hallmarks” geneset (“h.all.v7.5.1.symbols.gmt”) was used to perform the GSEA analysis, pathways with adjusted p-value (p.adjust) < 0.5 were visualized, and the absolute normalized enrichment score (NES) > 1.5 was considered statistically significant. All bars corresponding to significant genesets were colored in the bar diagram, while others were represented in unsaturated color.

### 2.13 Myeloid and T cell abundance analysis of samples in the scRNA-seq

Due to the heterogeneity of sampling, comparing immune infiltration from a macroscopic perspective might introduce bias. Hence, a comparison of immune infiltration based on immune subtype might provide practical information. To explore the relationship between the proportion of NecroLRS-High cells and the abundance of each cell subtype of “Myeloid” or “T” cells in each sample, we first calculated the proportion (Ratio_i_) of NecroLRS-High cells in each sample (i represents each sample).


$$Ratio_{i}=\frac{N_{NecroLRS-High_{i}}}{N_{NecroLRS-High_{i}}+N_{NecroLRS-Low_{i}}}$$

Then, the proportion of cell subtypes was visualized using a stacked-bar diagram and sorted based on the Ratio_i_. For further exploration of the correlation between Ratio_i_ and the proportion of specific cell subtypes, a scatter plot with a regressed line was utilized for visualization, and the Pearson (for Gaussian distribution) as well as Spearman (for abnormal distribution) correlation analysis was performed to determine the strength of the correlation.

### 2.14 Cell trajectory analysis

For further exploration of the developmental trajectory across non-malignant, NecroLRS-High, and NecroLRS-Low epithelial cells, the pseudo-temporal analysis based on “Monocle2” was utilized (34). After defining cell progress based on genes that differ between cell types, the DDRTree algorithm was utilized to reduce the dimensionality. The cell trajectory was visualized based on the cell types. Further, we assumed that the non-malignant epithelial cells were in an earlier phase and set them as the root of the cell trajectory. A pseudo-time-based diagram was then conducted to visualize the cell trajectory.

### 2.15 Cell communication analysis

To explore the crosstalk between NecroLRS-related cells and immune cells, the “CellChat” package was utilized (35). Based on the strength and weight of the interaction, the chord diagram and scatterplot were constructed for visualization. For further delineation of novel receptor-ligand pairs, signaling pathways that showed significant communication were retrieved and further visualized using the chord diagram and heatmap.

## 3 Results

### 3.1 Acquisition of necroptosis-correlated differential expressed lncRNAs

Differentially expressed lncRNAs between tumor and normal tissues were firstly acquired by differential expression analysis (|log2FC| > 1, adjusted P-value < 0.05, **Supplementary Table 4**). And the 145 necroptosis-related genes acquired previously were subjected to Pearson correlation analysis to determine necroptosis-correlated lncRNAs (|correlation coefficient| > 0.4, p < 0.001, **Supplementary Table 5**). Finally, a total of 519 lncRNAs were recognized as necroptosis-correlated differentially-expressed LncRNAs (DELncs) and utilized in downstream analysis.

### 3.2 Construction and validation of NecroLRS model

#### 3.2.1 NecroLRS model construction

**Figure 2:**
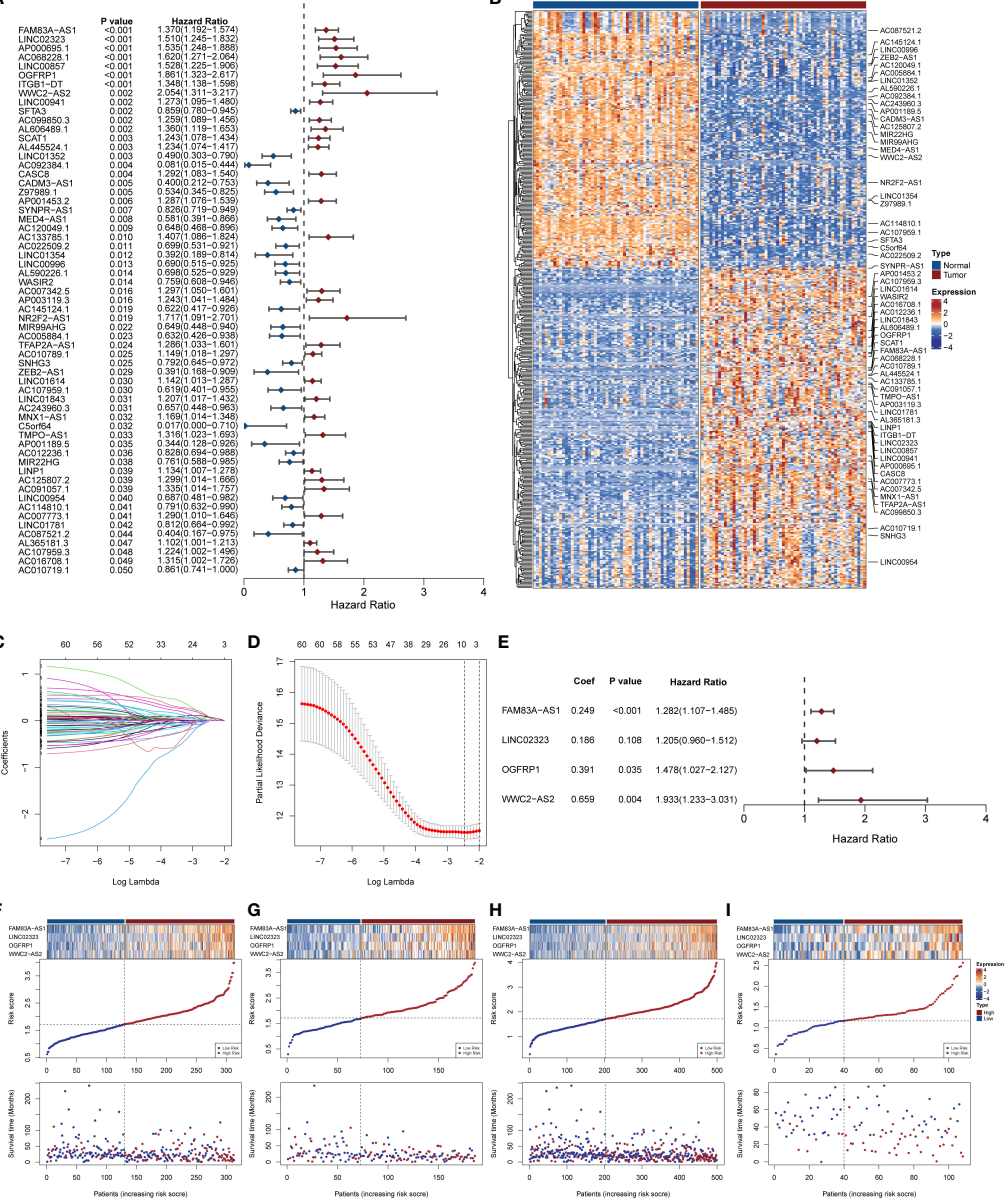
Extraction of necroptosis-related lncRNA signature and construction of Necroptosis-related LncRNA Risk Scoring (NecroLRS) model. **(A)** Prognostic lncRNAs extracted by univariate COX regression. The corresponding p-values and hazard ratios are listed following each lncRNA. The forest plot reveals the value and confidence interval of each lncRNA. The red color demonstrates corresponding lncRNA as a hazardous factor, while blue color indicates a protective factor. **(B)** Expression profile of prognostic lncRNAs extracted *via* univariate COX regression in matrices with paired TCGA-LUAD expression. Variable selection based on LASSO regression **(C)** and cross-validated errors of various levels of regularization **(D)** were visualized. **(E)** Necroptosis-related LncRNA Risk Scoring (NecroLRS) model information. **(F–I)** Exhibition of NecroLRS and corresponding patients’ survival status in training, test, whole, and validation cohorts.

Based on the expression matrix of 519 DELncs, the univariate Cox analysis was performed to determine survival-associated lncRNAs, and 61 DELncs were found to be significantly associated with overall survival (p < 0.05). The distribution of hazard ratios with corresponding P-value of these DELncs was presented as the forest diagram (**Figure 2A**). The gene expression profile in paired TCGA samples was visualized through a heat map (**Figure 2B**). To avoid overfitting, LASSO penalized regression was used, DELncs with zero coefficients were filtered out (**Figures 2C, D**), and those with non-zero coefficients were subjected to multivariate step-Cox regression analysis. Finally, four lncRNAs including FAM83A-AS1, LINC02323, OGFRP1, and WWC2-AS2 were selected to construct the NecroLRS model (**Figure 2E**), and the following formula was used to calculate the NecroLRS:


$$\begin{array}{c} NecroLRS\;=0.2486147\times {\left[FAM83A-AS1\right]}_{{Log}_{2}(TPM+1)}\\ \;\;\;\;\;+0.1862710\times {\left[LINC02323\right]}_{{Log}_{2}(TPM+1)}\\ \;\;\;\;\;+0.3906980\times {\left[OGFRP1\right]}_{{Log}_{2}(TPM+1)}\\ \;\;\;\;\;+0.6592927\times {\left[WWC2-AS2\right]}_{{Log}_{2}(TPM+1)} \end{array}$$

**Figure 3:**
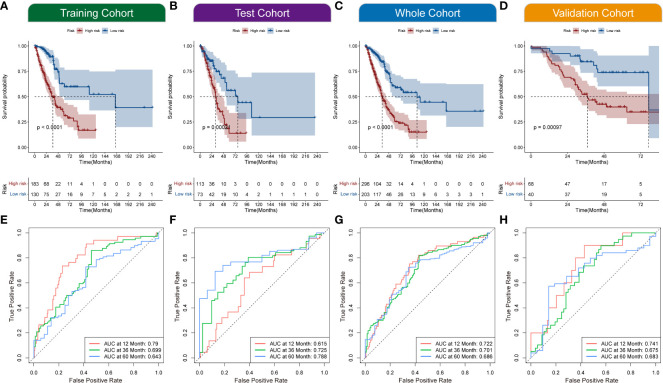
Exploration of NecroLRS model. **(A–D)** Kaplan-Meier survival analysis of NecroLRS-High and -Low groups in training, test, whole, and validation cohorts. The 1-, 3-, 5-year ROCs **(E–H)** of training, test, whole, and validation cohorts.

All the patients with LUAD were eventually classified into NecroLRS-High and NecroLRS-Low groups based on the cut-off values as described before. And the association between the expression profile of four LncRNAs and the distribution of survival states between the NecroLRS-High and NecroLRS-Low groups in the training, test, whole, and validation cohorts were shown in **Figures 2F–I**. Kaplan-Meier analysis of the survival trends showed that the NecroLRS-High group had a significantly worse survival than the NecroLRS-Low group (**Figures 3A–D**). Moreover, to evaluate the sensitivity and specificity of the model, a time-dependent ROC was used in all the four cohorts. The areas under the curve (AUC) associated with 1-, 3-, and 5-year survival rates were 0.79, 0.699, and 0.643, respectively, in the training cohort; 0.615, 0.725, and 0.788, respectively, in the test cohort; 0.722, 0.701, and 0.686, respectively in the whole cohort; and 0.741, 0.675, and 0.683 in the validation cohort, respectively (**Figures 3E–H**). Further, regardless of the TCGA or GEO cohort, the NecroLRS model showed good consistency and objectivity, indicating its strong stability (**Supplementary Figures 1A–H**). In summary, these results suggest that the NecroLRS model was a promising biomarker to predict the prognosis of patients with LUAD.

#### 3.2.2 Unsupervised clustering revealed favorable conformance across NecroLRS-related lncRNA profile and NecroLRS-related patient groups

**Figure 4:**
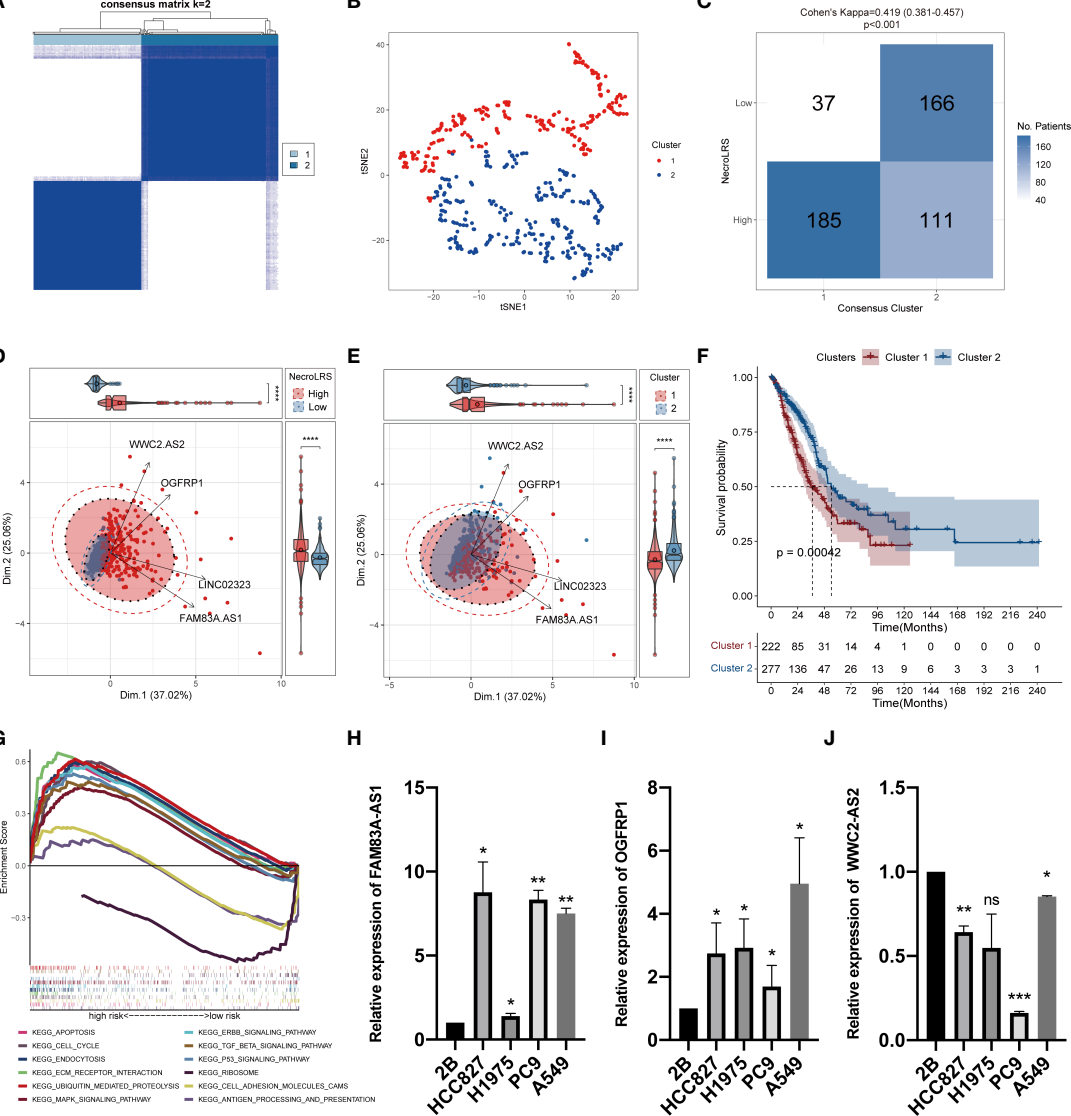
*In vitro* expression and unsupervised clustering based on NecroLRS-related lncRNAs. **(A)** Consensus matrix derived from ConsensusCluster algorithm (k = 2). **(B)** tSNE of 2 consensus clusters. **(C)** Confusion matrix revealed unsupervised clusters with good conformance shared with NecroLRS-related groups. **(D, E)** PCA analysis reveals efficient stratification of patients *via* consensus clustering and using NecroLRS model. **(F)** Kaplan-Meier survival analysis reveals poor prognosis of cluster 1. **(G)** KEGG enrichment analysis of NecroLRS-related groups. *In vitro* qRT-PCR validation of FAM83A-AS1 **(H)**, OGFRP1 **(I)**, and WWC2-AS2 **(J)**. *P < 0.05; **P < 0.01; ***P < 0.001; ****P < 0.0001; ns, P≥0.05.

To further evaluate the phenotypical differences mediated by the NecroLRS-related lncRNAs, consensus clustering based on the four lncRNA expression profile was used to regroup patients into two clusters, with the consistency matrices (k = 2) as shown (**Figure 4A**). The t-distributed stochastic neighbor embedding (t-SNE) was used to identify the distribution of patients based on the expression profile of four NecroLRS-related lncRNAs. We found that all patients with LUAD were divided into two subtypes (**Figure 4B**). Furthermore, the conformance between NecroLRS-related patient groups and unsupervised clusters was illustrated using a confusion matrix, in which cluster 1 was dominated by NecroLRS-High patients, while cluster 2 was mainly composed of NecroLRS-Low patients. Cohen’s test showed that NecroLRS-related lncRNA profile and NecroLRS-related patient groups shared good conformance (Kappa = 0.419, p < 0.001, **Figure 4C**). Next, we used principal component analysis (PCA) to project all tumor patients on the two-dimensional axis. Based on the top two principal components (PC), we found that both grouping methods were effective in differentiating patients and compatible with the previous confusion matrix (**Figures 4D–E**). In addition, cluster 1 showed a poor survival similar to the Kaplan-Meier analysis (**Figure 4F**). Taken together, regardless of the distribution of patients or overall survival, cluster 1 was similar to the NecroLRS-High group, while cluster 2 resembled the NecroLRS-Low group.

#### 3.2.3 Functional enrichment validation and gene set enrichment analysis

To validate the biological rationale of the NecroLRS model, we back-traced the four lncRNA-related genes to the original necroptosis-related gene list and performed GO as well as KEGG enrichment analysis to analyze potential selection biases. Results demonstrated that necroptosis-related pathways were highly enriched (**Supplementary Figures 2A, B**), which further reinforced the reliability of the necroptosis-related gene list initially acquired. In addition, based on the expression profile, GSEA analysis was performed to assess the enrichment of KEGG pathways in both NecroLRS-High and -Low subtypes (**Figure 4G**). Finally, we identified the nine KEGG pathways highly enriched in the NecroLRS-High subgroup, including apoptosis, cell cycle, endocytosis, ubiquitin-mediated proteolysis, ECM receptor interaction, TGF-beta, P53, ERBB, and MAPK signaling pathways, while three pathways were highly enriched in NecroLRS-Low subgroup, including antigen processing and presentation, cell adhesion molecules, and ribosome. We found that the pathways correlated with the NecroLRS-High group were mainly involved in apoptosis, tumor proliferation, progression, and metastasis, while the NecroLRS-Low group was mainly involved in immunity and cell development.

#### 3.2.4 Validation of lncRNAs expression by qRT-PCR

We detected the levels of four risk lncRNAs of the NecroLRS model in BEAS-2B, H1975, HCC827, A549, and PC9 cell lines using qRT-PCR. FAM83A-AS1 and OGFRP1 were highly expressed in LUAD cell lines (**Figures 4H–I**) compared to the BEAS-2B cell line, while WWC2-AS2 were poorly expressed in LUAD cell lines (**Figure 4J**). The LINC02323 was barely expressed in the LUAD cell lines we used in the current study. The results were generally consistent with the TCGA results (**Supplementary Figure 3**), which indicates the credibility of our bioinformatics analysis.

### 3.3 Immune infiltration analysis revealed a novel immune landscape in bulk-seq

**Figure 5:**
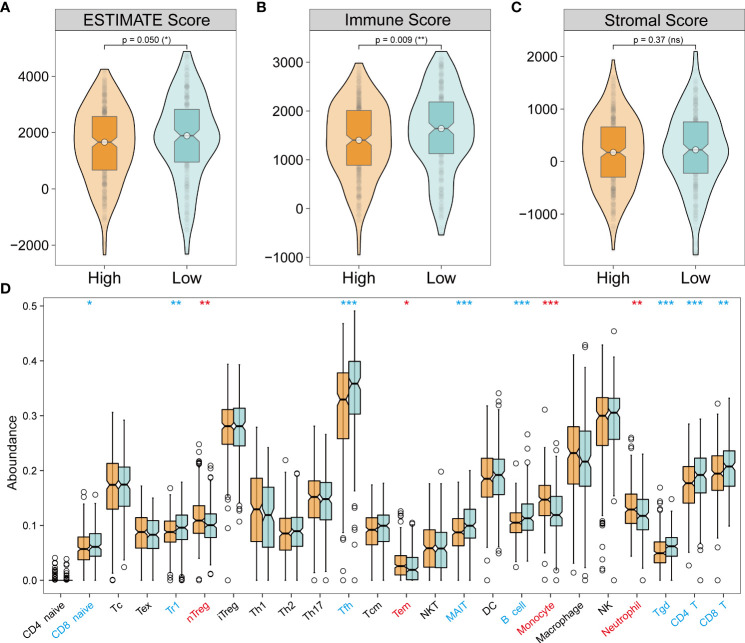
Tumor immune landscape of NecroLRS-High and -Low groups. **(A–C)** ESTIMATE algorithm reveals that the NecroLRS-High group has a lower ESTIMATE and immune score compared with the NecroLRS-Low group. **(D)** ImmuneCellAI algorithm reveals immune cell abundance across NecroLRS-High and -Low groups. *P < 0.05; **P < 0.01; ***P < 0.001; ns, P≥0.05.

Analysis of tumor immune microenvironment (TIME) revealed varying composition and proportion of tumor-infiltrating immune cells suggesting the potential mechanism of different pathways mediating tumorigenicity. Interactions between immune cells and other cells determine the strength of the anti-tumor immunity and were closely related to tumor progression and clinical prognosis (36–38). Therefore, it is essential to explore the necroptosis-mediated TIME heterogeneity in LUAD. Based on ESTIMATE analysis, which was used to evaluate the immune infiltration across the NecroLRS-related groups, the NecroLRS-High group had lower ESTIMATE and immune scores (**Figures 5A–C**). In addition, ImmuneCellAI analysis was performed and a majority of naturally occurring regulatory T (nTreg) cells, effector-memory T (Tem) cells, monocytes, and neutrophils were found to have a higher abundance in NecroLRS-High patients (**Figure 5D**). To summarize, the NecroLRS-High group showed an immunosuppressed tumor microenvironment with a higher infiltration of immunosuppressive cells, whereas the NecroLRS-Low group exhibited increased immune cell infiltration.

**Figure 6:**
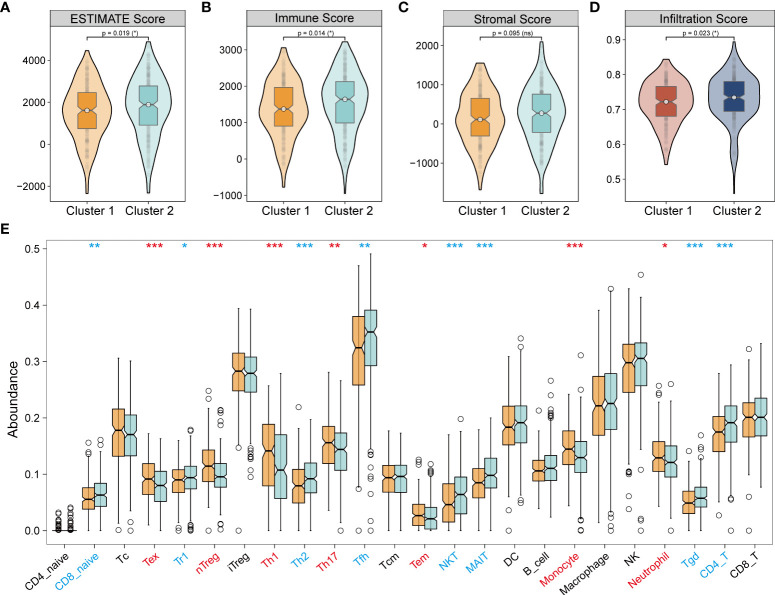
Tumor immune infiltration in unsupervised clusters. **(A–C)** ESTIMATE algorithm reveals that cluster 1 has a lower ESTIMATE and immune score compared with cluster 2. **(D)** ImmuneCellAI algorithm reveals that cluster 1 has lower immune infiltration compared with cluster 2. **(E)** ImmuneCellAI algorithm reveals immune cell abundance across clusters 1 and 2 (Red refers to significantly highly expressed in clusters 1, blue refers to significantly highly expressed in clusters 2). *P < 0.05; **P < 0.01; ***P < 0.001; ns, P≥0.05.

In the subgroups of unsupervised clusters, the ESTIMATE and ImmuneCellAI algorithms were used to assess immune infiltration. Cluster 2 showed higher immune infiltration compared with cluster 1 (**Figures 6A–D**). The composition of immune cells across the clusters (**Figure 6E**) revealed that naive CD8^+^ T cells, T regulated (Tr1) cells, T helper 2 (Th2) cells, follicular helper T (Tfh) cells, NKT cells, MAIT cells, Tgd cells, and CD4^+^ T cells were abundantly distributed in cluster 2, while Tex cells, Treg cells, nTreg cells, T helper 1 (Th1) cells, T helper 17 (Th17) cells, Tem cells, monocytes, and neutrophils were more abundant in cluster 1. Emerging studies have proven that Treg cells, Tex cells, and myeloid-derived suppressor cells (MDSC) mediate resistance to immunotherapy and exert a pro-tumorigenic effect (39–43). In our study, cluster 1 showed immune infiltration of Treg cells, Tex cells, monocytes, and neutrophils, which indicated that cluster 1 was more inclined to a cold tumor phenotype, while cluster 2 resembled a hot tumor phenotype characterized by infiltration of T cells. Taken together, consensus clusters further complemented the potential immune infiltration landscape of NecroLRS-related groups.

In general, we analyzed the immune infiltration environment of NecroLRS-related groups and clusters. To narrow the target immune cells, we acquired the co-existed immune patterns in both the NecroLRS-High group and cluster1 by intersecting the highly infiltrated immune cells in these two groups. Thus, we focused our attention on neutrophils, nTreg cells, Tem cells, and monocytes, and they were subjected to downstream analyses. Recent evidence suggested that neutrophils not only exhibited an anti-tumor effect mediated *via* chemotactic function but also promoted tumor invasion, metastasis, angiogenesis, and extracellular matrix remodeling by inhibiting anti-tumor immune surveillance. Accordingly, the characteristic distribution of neutrophils between NecroLRS-High and -Low groups is of crucial importance.

### 3.4 NecroLRS-related cell subgroups reveal distinct developmental trajectories

**Figure 7:**
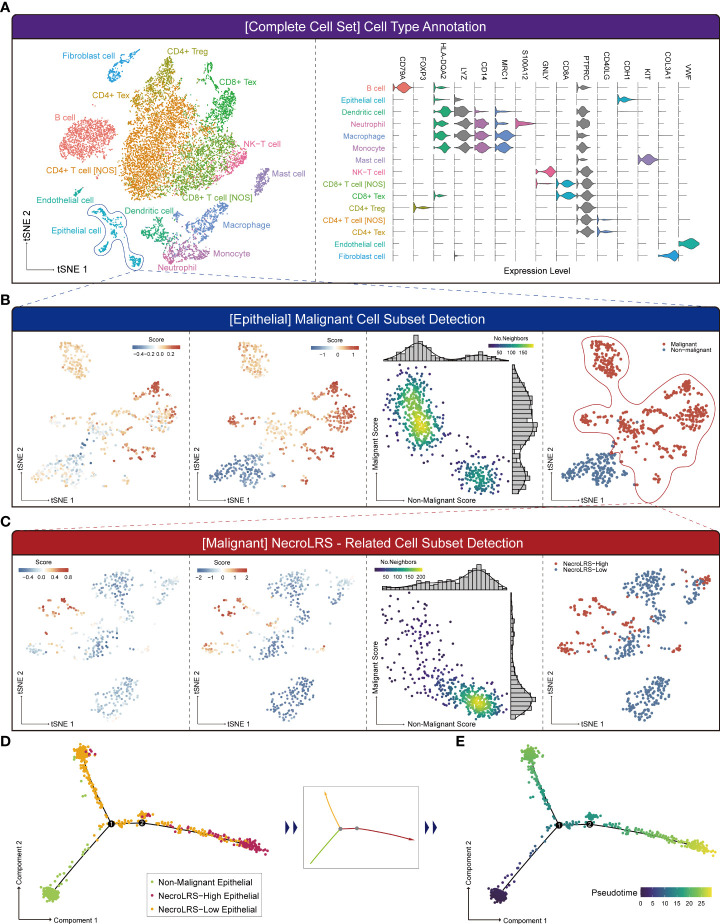
Single-cell RNA-seq-based NecroLRS-related subcluster exploration. **(A)** tSNE of the 13989 cells derived from 6 patients diagnosed with lung adenocarcinoma (left); based on cell type annotation complete cell sets are categorized into 15 cell subtypes, and representative markers of cell types are visualized using a violin plot (right). **(B)** Analysis of epithelial cell subset using malignant cell detection algorithm. The first figure reveals the initial malignant score of each cell. Following iterations, the scores and markers across malignant and non-malignant cell subsets reached a plateau (second figure). The k-means distributions of both malignant and non-malignant scores were visualized *via* scatter plot (third figure). Each point corresponds to a cell and is color-coded to indicate the number of neighbors, which reflects the density. Finally, malignant and non-malignant cell subsets were obtained based on the malignant and non-malignant scores. **(C)** Analysis of malignant cell subset using NecroLRS-associated cell detection algorithm. Cell trajectory of epithelial cells, grouped according to NecroLRS-associated cell subsets **(D)**. Cell trajectory of epithelial cells, based on pseudo-time assumed in the condition of non-malignant epithelial subcluster as the root of trajectory **(E)**.

Based on the transcriptome profiling, we observed a distinct functional difference between NecroLRS groups in bulk-seq. To elucidate the potential cell transition, pseudo-temporal analysis was performed based on the results of classification of the K-means classifier. In the current study, we reannotated a LUAD single-cell sequencing dataset (**Figure 7A** and **Supplementary Figure 4A**) and acquired the epithelial subset for the next-step analyses. According to the previously described algorithm, epithelial cells were classified into “Non-malignant” and “Malignant” cells, while “Malignant” cells were further classified into “NecroLRS-High” and “NecroLRS-Low” cell groups (**Figures 7B, C**). Based on the three cell subtypes, we utilized the Monocle2 toolkit for trajectory analysis. The results revealed a distinct trajectory of transition across cell subgroups of epithelial cells (**Figure 7D**). Further, we assumed that the “Non-malignant” epithelial cells were in an earlier phase in the trajectory and performed a pseudotime assignment. The pseudotime trajectory axis indicated that “Non-malignant” epithelial cells transdifferentiated into both “NecroLRS-High” and “NecroLRS-Low” cells (**Figure 7E**). Interestingly, “NecroLRS-High” cells might be located in a more terminal phase than “NecroLRS-Low” cells. These results indicate a potential transition of epithelial cells from a single-cell perspective for the first time and generation of NecroLRS-related cell subgroups *via* distinct developmental trajectories.

### 3.5 NecroLRS-High subset associated with inflammatory microenvironment and aggressive tumorigenic phenotype

The KEGG analysis of bulk-seq revealed a significant enrichment of apoptosis-related, ubiquitin-mediated proteolysis, ECM receptor interaction, and MAPK, ERBB, TGF-β, and p53 signaling pathways in NecroLRS-High groups, which indicated a significant heterogeneity between NecroLRS-High and -Low groups. To elucidate the biological function as well as immune infiltration heterogeneity between these two groups, GO, KEGG, and GSEA analyses were performed in the scRNA-seq.

**Figure 8:**
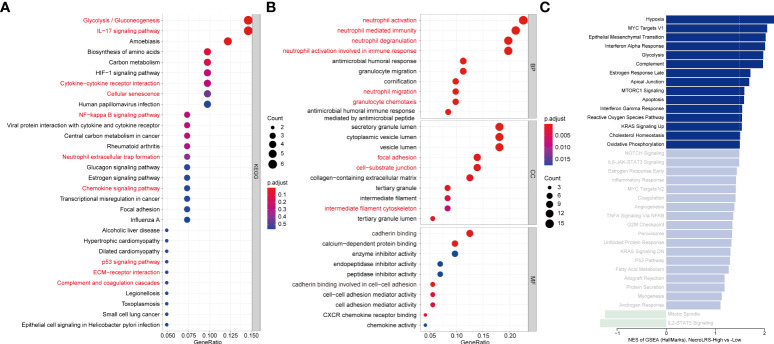
Single-cell RNA-seq-based enrichment analysis. **(A)** KEGG analysis based on marker genes in the NecroLRS-High subcluster. **(B)** GO analysis based on marker genes in the NecroLRS-High subcluster. **(C)** GSEA analysis based on differentially expressed marker genes among NecroLRS-related subclusters. Bars corresponding to pathways that meet absolute NES > 1.5 are colored in the bar diagram, while others are shown in unsaturated color.

In the KEGG analysis, cellular senescence, p53 signaling, ECM receptor interaction, inflammation-related pathways including IL-17 signaling, NF-kappa B signaling, neutrophil extracellular trap formation, complement and coagulation cascades, cytokine-cytokine receptor interaction, and chemokine signaling pathways were more activated in the NecroLRS-High cell subset (**Figure 8A**), which was in accordance with the results of bulk-seq. In GO analysis, neutrophil-related biological processes, chemotaxis of immunocytes, cytoskeleton-related cellular components, and molecular functions were highly enriched in the NecroLRS-High cell subset (**Figure 8B**). In GSEA analysis, a total of 15 hallmarks pathways including hypoxia, epithelial-to-mesenchymal transition (EMT), IFN-alpha and -gamma response, apoptosis, complement, and KRAS signaling pathways were enriched in the NecroLRS-High cell subset (**Figure 8C**).

Taken together, in the NecroLRS-High cell subset, the activity of apoptosis and cellular senescence was higher than in NecroLRS-Low cells. Functional enrichment of inflammatory mechanisms further indicated that NecroLRS-High cells might be accompanied by an inflammatory microenvironment. Additionally, cytoskeleton-related pathways and EMT pathways were also up-regulated, which indicated a more aggressive tumorigenic phenotype in NecroLRS-High cells and explained the distinct clinical outcomes between NecroLRS-High and -Low patients.

### 3.6 NecroLRS-High ratio influences neutrophil enrichment and T cell exhausting

**Figure 9:**
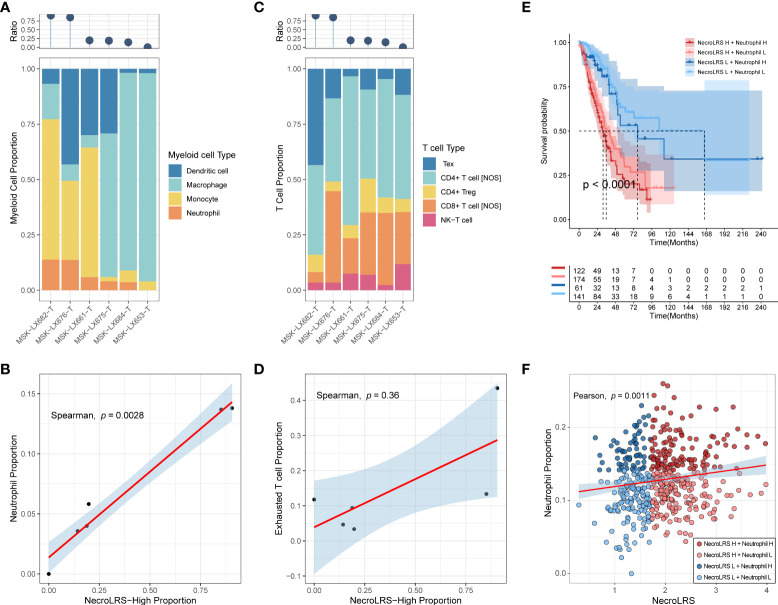
Immune abundance analysis of NecroLRS-related cell subgroups. The proportion of myeloid **(A)** and T **(C)** cell subtypes were visualized using stacked-bar diagrams and sorted according to the proportion of NecroLRS-High cells in malignant cells. **(B)** Correlation between the proportion of NecroLRS-High cells among malignant cells and the proportion of neutrophils among the myeloid cells. **(D)** Correlation between the proportion of NecroLRS-High cells among malignant cells and the proportion of exhausted T cells. **(E)** Survival analysis across different permutations of NecroLRS- and neutrophil-related patient groups in bulk-seq (TCGA whole cohort). **(F)** Correlation between NecroLRS and the proportion of neutrophils based on ImmuneCellAI algorithm in bulk-seq (TCGA whole cohort).

As the results of the previous analysis, we reported that NecroLRS-High patients always showed a higher proportion of myeloid cell infiltration in the TME. Further, we explored the infiltration of myeloid subtypes in scRNA-seq. As previously described, we acquired the NecroLRS-High cell proportion (Ratio_i_) in each sample and calculated the relative proportion of each myeloid cell type. Consistent with the findings of bulk-seq, we found that as Ratio_i_ increased, the proportion of neutrophils increased simultaneously (**Figure 9A**). The correlation analysis (**Figure 9B**) further demonstrated the proportion of NecroLRS-High cells was correlated with the proportion of neutrophils in myeloid cells (p = 0.0028). Further, the proportion of monocytes was high in patients with elevated Ratio_i_, although no significant correlation existed between the proportion of NecroLRS-High cells and the proportion of monocytes. This phenomenon suggested emerging interactions between malignant epithelial and myeloid cells during necroptosis.

Tex cells received increasing attention in recent years. Similar to myeloid cells, we also annotated Tex cells using markers reported previously (CTLA-4, LAG-3, TIGIT), and found that as Ratio_i_ increases, the proportion of Tex cells increases (**Figure 9C**). However, there was no significant correlation detected between the proportion of NecroLRS-High cells and the proportion of Tex cells in T cells (p = 0.36, **Figure 9D**). Similar to Tex, no numerical correlation was found between the proportion of other T cell subtypes and the proportion of NecroLRS-High cells.

It is well established that neutrophil infiltration is tightly associated with the progression of various tumors, including lung cancer. As previously described, we observed a potential association between neutrophil infiltration and NecroLRS in both bulk- and scRNA-seq. Further, we evaluated the prognostic ability of NecroLRS in combination with neutrophil infiltration based on ImmuneCellAI algorithm. The results showed a positive correlation between NecroLRS and relative neutrophil abundance (**Figure 9F**). Distinct survival outcomes were found under different permutations of NecroLRS and neutrophil conditions, in particular, among patients with high NecroLRS and neutrophil infiltration, who showed a relatively worse prognosis, while patients with low NecroLRS and neutrophil infiltration showed prolonged survival (**Figure 9E**).

### 3.7 Novel ligand-receptor pairs between immune cells and NecroLRS-High lung adenocarcinoma cells

Although NecroLRS-High patients showed a relatively lower level of immune infiltration in the bulk-seq, the abundance of different subtypes of immune cells differed distinctly between NecroLRS-High and -Low patients. Therefore, we further explored the communication between NecroLRS-related cell subsets and other immune cells in an effort to elucidate the differences in potential immune landscape between the two biological subtypes.

**Figure 10:**
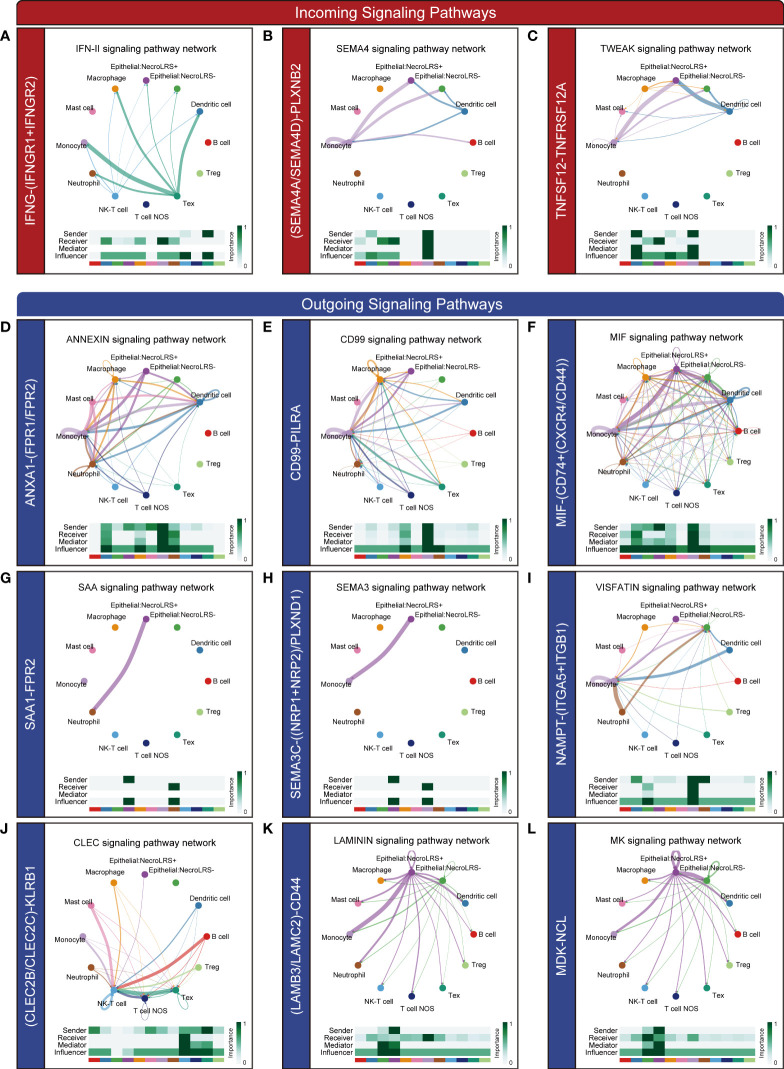
Novel ligand-receptor pairs differ between NecroLRS-High cells and immune cells. Ligand-receptor pairs in the incoming **(A–C)** and outgoing **(D–L)** interaction of NecroLRS-High cells with immune cells.

In addition to NecroLRS-related epithelial cells, we incorporated another ten immune subtypes including neutrophils, macrophages, dendritic, monocytes, mast, B, NK-T, Tex, Treg, and the remaining T cells (T cell NOS). According to the summary of incoming and outgoing cellular interactions, the NecroLRS-Low cell subset exhibited a higher strength of incoming interaction, while the NecroLRS-High cell subset revealed a stronger outgoing interaction (**Supplementary Figures 4B–D**). Compared with NecroLRS-Low cells, NecroLRS-High cells received the signal from Tex, monocytes, macrophages, and dendritic cells *via* the IFN-II (IFNG-(IFNGR1+IFNGR2), **Figure 10A**), SEMA4 (SEMA4A-PLXNB2, SEMA4D-PLXNB2, **Figure 10B**), and TWEAK pathways (TNFSF12-TNFRSF12A, **Figure 10C**). According to the outgoing signal perspective, NecroLRS-High cells transmit the signal to neutrophils, macrophages, monocytes, dendritic, B, and NK-T cells *via* the ANNEXIN pathway (ANXA1-FPR2, ANXA1-FPR1, **Figure 10D**), CD99 (CD99-PILRA, **Figure 10E**), MIF (MIF-(CD74+CXCR4), MIF-(CD74+CD44), **Figure 10F**), SAA (SAA1-FPR2, **Figure 10G**), SEMA3 (SEMA3C-(NRP1+NRP2), SEMA3C-PLXND1, **Figure 10H**), VISFATIN (NAMPT-(ITGA5+ITGB1), **Figure 10I**), and CLEC pathways (CLEC2B-KLRB1, CLEC2C-KLRB1, **Figure 10J**). Additionally, NecroLRS-High cells also transmit the signal to all the immune cells *via* the LAMININ pathway (LAMB3-CD44, and LAMC2-CD44, **Figure 10K**) and MK pathway (MDK-NCL, **Figure 10L**).

Taken together, NecroLRS-High cells communicated more actively with macrophages, monocytes, neutrophils, Tex, NK-T, B, and dendritic cells compared with NecroLRS-Low cells, which might explain the differences in immune infiltration between the two categories of NecroLRS cells.

### 3.8 Clinical utility of hybrid model

**Figure 11:**
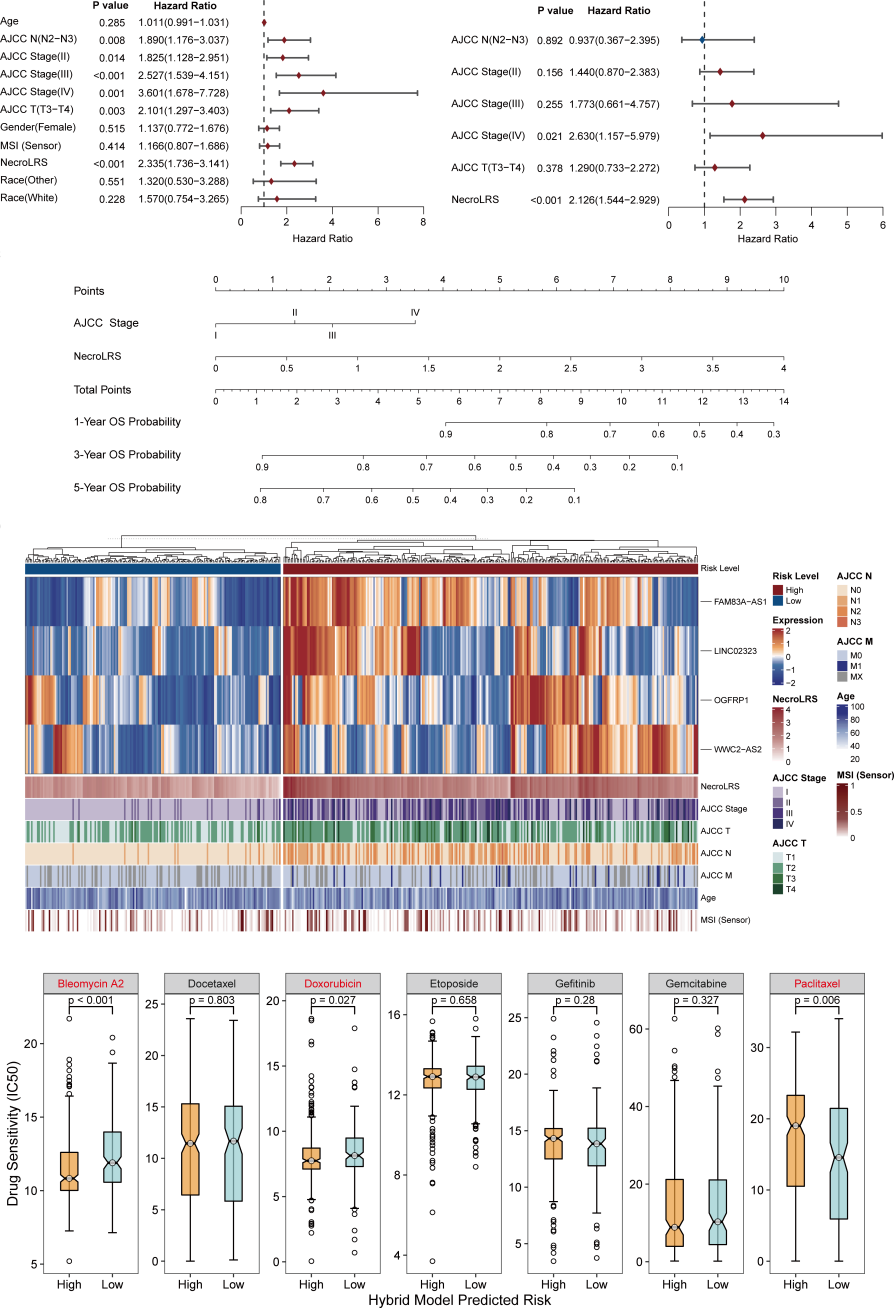
Extraction of clinicopathological signature and construction of necroptosis-related hybrid model. **(A)** Univariate COX analysis of all clinicopathological factors including NecroLRS. **(B)** Multivariate COX analysis of prognostic clinicopathological factors. **(C)** Nomogram of necroptosis-related hybrid Model. **(D)** NecroLRS-associated lncRNA expression and corresponding prognostic clinicopathological factors in high- and low-risk groups in the hybrid model. **(E)** The oncoPredict algorithm was used to determine drug sensitivity of seven different chemo- or immuno-therapeutic compounds.

To gain insight into the prognostic value of NecroLRS combined with clinicopathological variables, both univariate and multivariate Cox analyses were utilized to identify the independent prognostic factors. Following univariate Cox analysis, both AJCC N, AJCC stage, AJCC T, and NecroLRS were found to be associated with the survival of patients, and therefore further included in multivariate Cox analysis. AJCC stage and NecroLRS were independent prognostic factors (**Figures 11A, B**). Based on the independent prognostic factors, a hybrid model was constructed, and a nomogram was established to predict the prognosis of 1-, 3- and 5-year OS of patients with LUAD (**Figure 11C**). Next, patients were separated into high-risk and low-risk groups according to the hybrid model. The profile of four NecroLRS-related LncRNAs, NecroLRS, and clinicopathological factors across two groups was visualized through a heatmap. Patients in the high-risk group exhibited a higher NecroLRS and a more advanced AJCC Stage, as well as AJCC T, N, and M stages. (**Figure 11D**).

To evaluate the treatment sensitivity of patients in different subgroups based on the hybrid model, seven common chemotherapeutic and targeted drugs (“Bleomycin”, “Docetaxel”, “Doxorubicin”, “Etoposide”, “Gemcitabine”, “Gefitinib”, “ Paclitaxel”) were selected for drug sensitivity prediction. The low-risk group showed a higher IC_50_ for Bleomycin and Doxorubicin, suggesting that patients in the high-risk group exhibited higher sensitivity to Bleomycin and Doxorubicin, while the low-risk group patients were more likely to benefit from Paclitaxel (**Figure 11E**).

**Figure 12:**
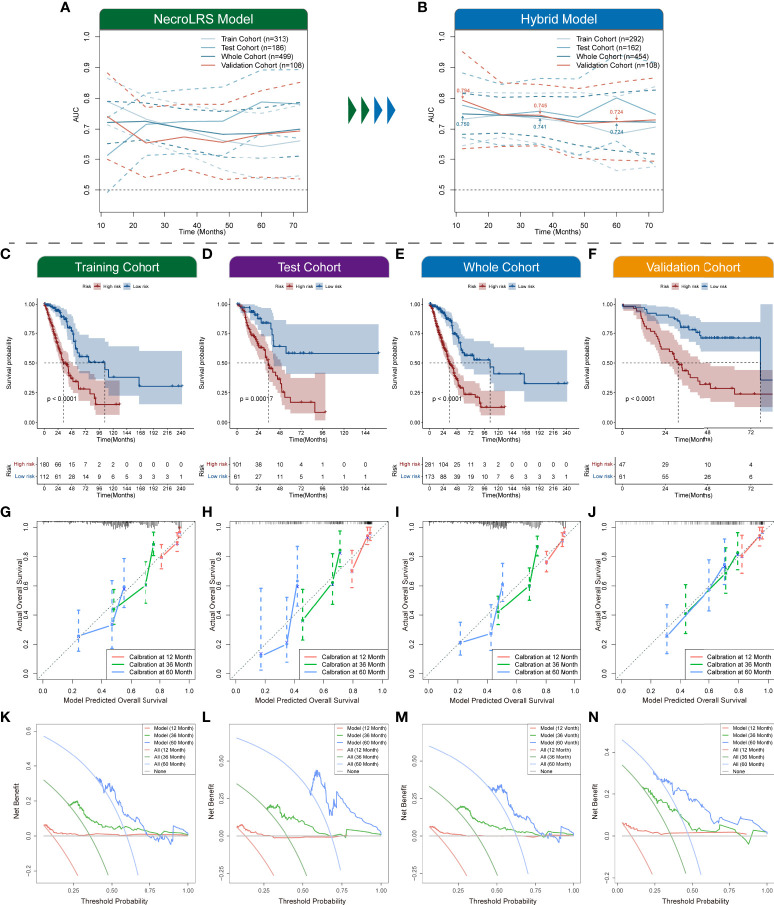
Exploration of necroptosis-related hybrid model. Time-dependent ROCs of both NecroLRS **(A)** and hybrid **(B)** model in training, test, whole, and validation cohorts. Additionally, the 1-, 3-, and 5-year AUC values of the hybrid model in the whole and validation cohorts were further annotated. **(C–F)** Kaplan-Meier survival analysis of high- and low-risk groups in training, test, whole, and validation cohorts, respectively. 1-, 3-, 5-year calibration curves **(G–J)** and DCA curves **(K–N)** of training, test, whole, and validation cohorts are shown (“None”: assume no patient will die at the specific time point and offer treatment to no one; “All”: assume all patients will die at the specific time point and therefore treat everyone; “Model”: gives the expected net benefit of hybrid model on each patient under different threshold probability).

To demonstrate the prognostic performance of the hybrid model, Kaplan-Meier analysis was used to compare the overall survival of patients in the high- and low-risk groups (**Figures 12C–F**). The time-dependent ROC profile demonstrated that the hybrid model exhibited a more robust and higher level of AUC compared with the NecroLRS model (**Figures 12A, B**). In addition, the calibration and DCA analyses were further performed (**Figures 12G–N**). The hybrid model showed superior predictive performance compared with the NecroLRS model, with higher sensitivity and specificity and additional clinical benefit. In general, these results revealed that the optimized hybrid model was a reliable tool to determine the prognosis of patients with LUAD.

## 4 Discussion

Investigation into the tumorigenicity of lung cancer revealed emerging pathways in the disease spectrum. However, there is a desperate need for favorable biomarkers to facilitate precision diagnosis as well as targeted treatment. As a novel form of programmed cell death that resembles both apoptosis and necrosis, necroptosis plays a pivotal role in cancer biology (44). In addition, lncRNA is a basic subtype of noncoding genomes, and plays a crucial role in tumorigenicity, considering its sponge-like biological function as well as diverse regulatory effects. LncRNA has been widely explored and proved to act as a promising biomarker in a variety of tumors. Hence, in the current study, we constructed a novel signature (NecroLRS) based on necroptosis-related lncRNAs. For clinical application, a hybrid model showed acceptable sensitivity and specificity was constructed. Application of the NecroLRS and hybrid models in both training, test, whole, and validation cohorts revealed a distinct survival difference in Kaplan-Meier analysis. Time-dependent ROC curves, calibration, and DCA further demonstrated their effectiveness in predicting the overall survival of patients with LUAD. Further, qRT-PCR was used to validate the expression of NecroLRS-related lncRNAs *in vitro*. For the exploration of the immune landscape, we used multiple algorithms to evaluate immune infiltration in NecroLRS subgroups. The unique distribution of neutrophils, nTreg, Tem, and monocytes attracted our attention due to their high infiltration in the NecroLRS-High group. Further, combined with single-cell sequencing, we investigated the developmental transition between epithelial cells and NecroLRS-related cell subgroups and explored the potential causes of the unique immune landscape in bulk-seq *via* cell communication and immune distribution analyses.

Prior studies revealed the pivotal biological effects of NecroLRS-related lncRNAs in a wide variety of tumors as well as diseases. For instance, FAM83A-AS1, as a risk factor, promoted LUAD progression by targeting miR-150-5p and ultimately promoting the expression of MMP14 (45). A multi-cohort study conducted by Wang et al. (Jiangsu Cancer Hospital and TCGA cohort) demonstrated that FAM83H-AS1 enhanced the expression of RAB8B and RAB14, thereby inhibiting apoptosis and facilitating tumorigenesis in LUAD cells (46). In addition, FAM83A-AS1 promoted the progression of esophageal squamous cell carcinoma (ESCC) by regulating the miR-214/CDC25B axis (47). LIN02323, as a novel lncRNA associated with EMT, has been proposed to mediate the metastasis of LUAD *via* the miR-1343-3p/TGFBR1 axis (48). OGFRP1 acts as an oncogene to promote the development and progression of many kinds of tumors including lung, colon, gastric, and prostate cancers (49–52). Although WWC2-AS2 has been mentioned as one of the favorable prognosis-related predictors of LUAD by Lu et al, the underlying mechanism of WWC2-AS2 in the prognosis of LUAD remains to be further investigated (53).

It is reported that necroptosis mediates the differential response to immunotherapy in a variety of cancers by affecting the TME, suggesting its role as a promising indicator in cancer diagnosis as well as treatment. However, the underlying dynamics of altered immune infiltration landscape was poorly investigated. In this study, we assessed the relationship between necroptosis and the immune landscape of LUAD based on bulk-seq, which indicated that the NecroLRS-High patients exhibited a highly immunosuppressive phenotype and a higher infiltration of neutrophils, nTreg cells, Tem cells, and monocytes. The TME exhibits a “convolutional” pattern due to its highly cross-linked constituents. To investigate the immune infiltration, several algorithms were introduced to reveal the potential immune infiltration status. However, these algorithms only act as useful references instead of precision quantity tools. Therefore, to address this limitation, a single-cell-based approach was introduced in this study, which revealed the potential correlation between the proportion of NecroLRS-related cells and neutrophils in myeloid cells. Emerging evidence indicates that tumor-associated myeloid cells (TAMCs) including monocytes, tumor-associated macrophages (TAMs), dendritic cells (DCs), tumor-associated neutrophils (TANs), and myeloid-derived suppressor cells (MDSCs) play a pivotal and even contrasting role in tumor progression, and “double-edged” biological function also attracted increasing attention (54). In both bulk- and scRNA-seq, we observed the unique distribution pattern of neutrophils, which indicated a potential correlation between neutrophils and necroptosis. According to previous studies, neutrophils were tightly associated with the progression of various tumors including lung cancer (55, 56). In particular, TANs were polarized into 2 subtypes (N1 and N2) based on a variety of environmental factors including cytokines and chemokines in the TME, and the N1 TANs exhibited tumor suppression *via* activation of CD8+ T cells, while the N2 neutrophils stimulated tumor growth, invasion, metastasis, and angiogenesis (57). In this study, patients with higher NecroLRS showed a worse prognosis, which might be associated with N2 TANs. However, due to the limited sample size in scRNA-seq, further studies are needed. Furthermore, several studies have revealed that the activation of neutrophils facilitated the process of necroptosis, such as the presence of monosodium urate (MSU) crystals or adhesion receptors, which induced activation of the RIPK3-MLKL complex (58–60). However, these studies only focused on the inflammatory response driven by infection or autoimmune disorders, while the interaction between necroptosis and neutrophils in the tumor was rarely mentioned. Tex cells attracted increased attention in recent years as they were found not only in CD8+T cells but also in CD4+T cells (61, 62). According to Jiang et al., Tex overexpressed inhibitory receptors, decreased cytolytic activity, and ultimately resulted in decreased anti-cancer response (43). Based on the unsupervised clustering of NecroLRS-related lncRNAs’ expression profile, patients in cluster 1 showed a higher infiltration of Tex compared with those in cluster 2, while no significant difference in Tex infiltration was detected in the NecroLRS-related groups. In the scRNA-seq, similarly, no significant correlation was found between the proportion of NecroLRS-High cells and Tex cells, whereas patients with high NecroLRS-High cell population tended to show a high infiltration of Tex, which might partly explain the worse prognosis of NecroLRS-High patients. Overall, the scRNA-seq results suggest a potential cause of poor prognosis in NecroLRS-High patients and provide a reference for further research into the role of necroptosis in tumor development.

Previous studies have stressed that necroptosis triggers inflammatory responses and promotes immunosuppression (5, 63). In this study, we observed a similar phenomenon. As the damage-associated molecular patterns (DAMPs) generated during necroptosis, both antigens and inflammatory cytokines were released into the TME, which activated both innate and adaptive immune responses and resulted in tumor elimination. However, DAMPs also recruit inflammatory cells and promote tumor progression. In both bulk- and scRNA-seq, we found that high NecroLRS patients always manifested an inflammatory TME as well as inflammatory immune cell infiltration, which further explained the poor prognosis of this group of patients. In addition, hypoxia as a crucial mediator of necroptosis has been reported (64). The GSEA analysis of scRNA-seq ranked the hypoxia-related pathway as the most significant in the NecroLRS-High group of patients. Two crucial pathways including NF-Kappa B and glycolysis that are closely associated with hypoxia were also detected, which further implied the potential mechanism of necroptosis induction in LUAD (65).

Another notable feature of this study is that scRNA-seq was used to investigate the potential ligand-receptor pairs among immune cells and NecroLRS-High cells. Some of the pathways including the TWEAK pathway (TNFSF12-TNFRSF12A), ANXA1-FPR1, and LAMC2-CD44 have been reported previously in lung disease, which play a pivotal role in inflammatory immune mechanisms and immunosurveillance (66–68). Additionally, several novel ligand-receptor pairs were reported first in this study, including LAMB3-CD44, ANXA1-FPR2, IFN-II (IFNG-(IFNGR1+IFNGR2)), SEMA4 (SEMA4A-PLXNB2, SEMA4D-PLXNB2), CD99 (CD99-PILRA), MIF (MIF-(CD74+CXCR4), MIF-(CD74+CD44)), SAA (SAA1-FPR2), SEMA3 (SEMA3C-(NRP1+NRP2), SEMA3C-PLXND1), VISFATIN (NAMPT-(ITGA5+ITGB1)), CLEC (CLEC2B-KLRB1, CLEC2C-KLRB1), and MK pathways (MDK-NCL). The role of these ligand-receptor pairs has been partly demonstrated in other diseases as well as cancers; however, their potential roles in necroptosis-induced tumorigenesis of LUAD cannot be disregarded. Studies revealed that these pathways play a crucial role in the interaction between malignant cells and TIME. Further studies focused on these pathways might elucidate the underlying mechanism of necroptosis-associated tumorigenesis and provide promising targets for the treatment of patients with LUAD.

There were already necroptosis-related models developed in other cancers. For instance, in hepatocellular carcinoma, consistent with our result, the higher necroptosis risk patients had shorter overall survival and lower efficiency of immunotherapies (69). In addition, a cell necroptosis index (CNI) was established in triple-negative breast cancer (TNBC) and exactly showed that the poor prognosis of TNBC patients with high-CNI was linked to chemotherapy resistance (70). Both studies have shown the promising prognostic significance of necroptosis-related patterns. Although a necroptosis-related lncRNA signature has been reported recently, the current study has shown unique strengths from several perspectives. In contrast with the results reported by Lu et al. (71), our study provides a more comprehensive workflow based on a model established for cross-cohort validation. Our study shows superior model performance as well as clinical utility. In the current study, the AUCs of the hybrid model used to predict 1-, 3-, and 5-year OS rates were 0.75, 0.741, and 0.724, respectively, while in Lu’s study the AUCs were 0.723, 0.679, and 0.715, respectively. Due to the defects in study design, the study of Lu et al. lacks external validation. By contrast, our study was validated both internally and externally, and the AUCs of the hybrid model predicting 1-, 3-, and 5-year OS rates were 0.794, 0.745, and 0.724, respectively, suggesting the robustness of our findings. Further, our study involved a systematic evaluation of the performance of each model, and the prognostic performance of the models was validated in an independent cohort using the same sequencing technique as the training cohort, which further verified the robustness and generalizability of our findings. In addition, qRT-PCR was used in the current study to validate the NecroLRS-related lncRNAs’ expression in LUAD cell lines, the expression of OGFRP1 and FAM83A-AS1 was higher than in BEAS-2B cell line, while WWC2-AS2 was poorly expressed in LUAD cell lines than in BEAS-2B cell line, which further corroborated our bioinformatics results. The LINC02323 was barely expressed in LUAD cell lines we used in the current study, however, Zhang et al. (48) have demonstrated that LINC02323 was highly expressed in the SPC‐A‐1 cell line and barely expressed in H1299, H1975, and A549 cell lines, which was generally consistent with the current study. Although WWC2-AS2 is lower expressed in tumor tissues, it may have potential biological functions. During the development of cancer, the molecules vary depending on the type and stage (72). For example, CHAC1 is down-regulated in kidney renal clear cell carcinoma (KIRC) samples when compared with normal samples, but in KIRC samples, a higher expression level was observed in patients with higher malignancy and later stages (73). The differential expression analysis is based on the tumor and normal patients, while the survival analysis is performed on inner tumor patients. Thus, differential expression and survival analysis should not be endowed with mathematical relationships. In our research, the expression pattern of WWC2-AS2 in different AJCC stages of LUAD patients was evaluated and an up-regulated expression of WWC2-AS2 was observed in the advanced stage, which explained why WWC2-AS2 could act as a risk factor in the current study to some extents. Further, we have investigated the immune infiltration landscape in both bulk and single cells. The cell communication analysis combined with immune analysis using scRNA-seq further reinforced the findings of bulk-seq. We elucidated the distinct developmental trajectories of NecroLRS-related cell subgroups apart from normal epithelial cells and established that neutrophils might serve a crucial role in necroptosis-related tumorigenesis of LUAD. Despite the novelty of our study, the sampling bias generated by tumor heterogeneity could not be eliminated, and the key molecules have not been experimentally validated by further functional validation and immune correlation analysis in the current study. In addition, the prediction model utilized a public cohort, and the retrospective nature limits the persuasiveness. Although the results of ROC analysis are superior to similar published studies, there are still few variables in the mixed model and further validation by prospective studies with a large sample number is needed before clinical application.

In summary, we proposed NecroLRS which constitutes 4 necroptosis-related lncRNAs as a promising prognostic predicting approach in LUAD. Our study revealed that NecroLRS was positively correlated with neutrophil enrichment, inflammatory immune response, and malignant phenotypes of LUAD. Moreover, combining bulk- and scRNA-seq has revealed the developmental trajectory transition from nonmalignant epithelial to NecroLRS-related cell subtypes for the first time, and novel ligand-receptor pairs also provide promising targets for future studies. In addition, a hybrid model that combines clinicopathological factors and NecroLRS showed superior prognostic performance, which represents a promising clinical tool.

## Data availability statement

The original contributions presented in the study are included in the article/**Supplementary Material**. Further inquiries can be directed to the corresponding authors.

## Author contributions

YY, TZ and DW conducted the experiments, LG and ZZ analyzed the data, YY, LG and ZZ wrote the manuscript. XX, LY, XH and LW contributed to the conception of the present research and was responsible for approving the version before publication. All authors participated in the design, manuscript revision, read, and approved the submitted version.

## Funding

This work was supported by the National Natural Science Foundation of China (82203322) and the Wenzhou science and technology project (H20210009 and H2020010). 

## Conflict of interest

The authors declare that the research was conducted in the absence of any commercial or financial relationships that could be construed as a potential conflict of interest.

## Publisher’s note

All claims expressed in this article are solely those of the authors and do not necessarily represent those of their affiliated organizations, or those of the publisher, the editors and the reviewers. Any product that may be evaluated in this article, or claim that may be made by its manufacturer, is not guaranteed or endorsed by the publisher.

## Supplementary material

Supplementary Figure 1 Evaluation of NecroLRS model. Calibration curves **(A–D)**, DCA curves **(E–H)** of training, test, whole, and validation cohorts (“None”: assume no patient will die at the specific time point and offer treatment to no one; “All”: assume all patients will die at the specific time point and therefore treat everyone; “Model”: gives the expected net benefit of NecroLRS model on each patient under different threshold probability.

Supplementary Figure 2 Bulk RNA-seq-based enrichment validation. GO **(A)** and KEGG **(B)** analysis of NecroLRS-related genes in the original necroptosis-related gene list.

Supplementary Figure 3 Expression profile of four risk lncRNAs in the NecroLRS model. The expression profile of FAM83A-AS1 **(A)**, OGFRP1 **(B)**, LINC02323 **(C)**, and WWC2-AS2 **(D)** between normal and tumor tissues in the TCGA cohort. The expression pattern of WWC2-AS2 in different subgroups of AJCC stages **(E)**.

Supplementary Figure 4 Cell annotation in single-cell RNA-seq analysis and cell communication landscape of NecroLRS-associated subclusters. **(A)** Expression of key cell type markers in each cell type after annotation. **(B)** The landscape of incoming and outcoming interaction strength across each cell cluster. **(C, D)** Strength of cell communication among NecroLRS-Low and NecroLRS-High subclusters.
