# Supplementary material for: Necroptosis-related lncRNAs: Combination of bulk and single-cell sequencing reveals immune landscape alteration and a novel prognosis stratification approach in lung adenocarcinoma
Source: Front Oncol. 2022 Oct 20;12:1010976. doi: 10.3389/fonc.2022.1010976 (PMC9808398; doi:10.3389/fonc.2022.1010976)
Supplement: Supplementary Figure 1 — Evaluation of NecroLRS model. Calibration curves (A–D), DCA curves (E–H) of training, test, whole, and validation cohorts (“None”: assume no patient will die at the specific time point and offer treatment to no one; “All”: assume all patients will die at the specific time point and therefore treat everyone; “Model”: gives the expected net benefit of NecroLRS model on each patient under different threshold probability. [file DataSheet_1.zip › Supplementary Table 2.docx]

Supplementary Table 2. The Baseline of Characteristics in TCGA and GEO Cohorts.

| **Variable** | **TCGA Cohort** | | | **GEO Cohort**  *(N = 108)*^1^ |
| --- | --- | --- | --- | --- |
|  | **Overall**  *(N = 499)*^1^ | **Test Cohort**  *(N = 186)^1^* | **Training Cohort**  *(N = 313)^1^* |  |
| **Gender** |  |  |  |  |
| Female | 269 (54%) | 93 (50%) | 176 (56%) | 69 (64%) |
| Male | 230 (46%) | 93 (50%) | 137 (44%) | 39 (36%) |
| **Age** | 66 (59, 72) | 67 (60, 73) | 65 (58, 72) | 68 (62, 74) |
| *(Missing)* | *21* | *5* | *16* | *0* |
| **Stage** |  |  |  |  |
| I | 268 (55%) | 103 (57%) | 165 (53%) | 62 (57%) |
| II | 119 (24%) | 35 (19%) | 84 (27%) | 19 (18%) |
| III | 79 (16%) | 35 (19%) | 44 (14%) | 24 (22%) |
| IV | 25 (5.1%) | 8 (4.4%) | 17 (5.5%) | 3 (2.8%) |
| *(Missing)* | *8* | *5* | *3* | *0* |
| **T** |  |  |  |  |
| T1 | 167 (33%) | 62 (33%) | 105 (34%) | - |
| T2 | 266 (53%) | 98 (53%) | 168 (54%) | - |
| T3 | 45 (9.0%) | 19 (10%) | 26 (8.3%) | - |
| T4 | 18 (3.6%) | 6 (3.2%) | 12 (3.8%) | - |
| TX | 3 (0.6%) | 1 (0.5%) | 2 (0.6%) | - |
| **N** |  |  |  |  |
| N0 | 324 (65%) | 122 (66%) | 202 (65%) | - |
| N1 | 94 (19%) | 31 (17%) | 63 (20%) | - |
| N2 | 68 (14%) | 27 (15%) | 41 (13%) | - |
| N3 | 2 (0.4%) | 1 (0.5%) | 1 (0.3%) | - |
| NX | 10 (2.0%) | 4 (2.2%) | 6 (1.9%) | - |
| *(Missing)* | *1* | *1* | *0* | *-* |
| **M** |  |  |  |  |
| M0 | 331 (67%) | 128 (70%) | 203 (65%) | - |
| M1 | 24 (4.8%) | 7 (3.8%) | 17 (5.5%) | - |
| MX | 140 (28%) | 49 (27%) | 91 (29%) | - |
| *(Missing)* | *4* | *2* | *2* | *-* |

^1^Median (IQR) or Frequency (%)
