# Supplementary material for: Necroptosis-related lncRNAs: Combination of bulk and single-cell sequencing reveals immune landscape alteration and a novel prognosis stratification approach in lung adenocarcinoma
Source: Front Oncol. 2022 Oct 20;12:1010976. doi: 10.3389/fonc.2022.1010976 (PMC9808398; doi:10.3389/fonc.2022.1010976)
Supplement: Supplementary Figure 1 — Evaluation of NecroLRS model. Calibration curves (A–D), DCA curves (E–H) of training, test, whole, and validation cohorts (“None”: assume no patient will die at the specific time point and offer treatment to no one; “All”: assume all patients will die at the specific time point and therefore treat everyone; “Model”: gives the expected net benefit of NecroLRS model on each patient under different threshold probability. [file DataSheet_1.zip › Supplementary Table 3.docx]

**Supplementary Table 3 Sequence of primers utilized in the current study.**

| Primer name | Sequence (5’-3’) |
| --- | --- |
| LINC02323 F | GTCAAGACATCCAGAAAG |
| LINC02323 R | AAGACAAGTCCCAGAGAG |
| OGFRP1 F | GAGTTTAACTTATCCCAA |
| OGFRP1 R | TCTTACATTCTTTCCCTA |
| FAM83A-AS1 F | TCGGTGCTATGAAGGACA |
| FAM83A-AS1 R | TAGGGTGGGGCAAGTTTT |
| WWC2-AS2 F | CGCCCCAGTCCATTCATC |
| WWC2-AS2 R | GGCAGGTCCCATCTTTTT |
| GAPDH F | GGACCTGACCTGCCGTCTAG |
| GAPDH R | GTAGCCCAGGATGCCCTTGA |
