# Supplementary figures and images for: Necroptosis-related lncRNAs: Combination of bulk and single-cell sequencing reveals immune landscape alteration and a novel prognosis stratification approach in lung adenocarcinoma
Source: Front Oncol. 2022 Oct 20;12:1010976. doi: 10.3389/fonc.2022.1010976 (PMC9808398; doi:10.3389/fonc.2022.1010976)

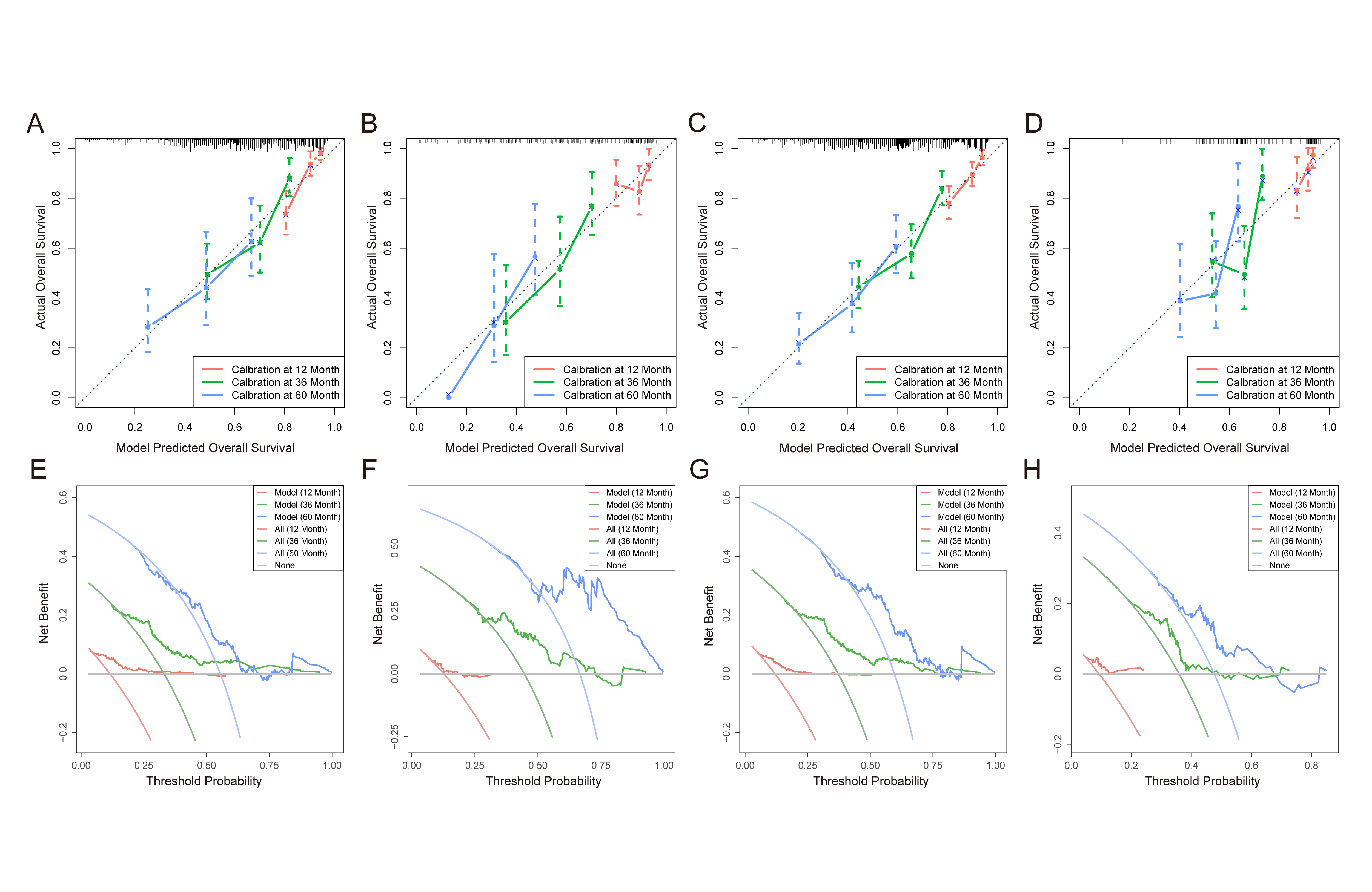

Supplement: Supplementary Figure 1 — Evaluation of NecroLRS model. Calibration curves (A–D), DCA curves (E–H) of training, test, whole, and validation cohorts (“None”: assume no patient will die at the specific time point and offer treatment to no one; “All”: assume all patients will die at the specific time point and therefore treat everyone; “Model”: gives the expected net benefit of NecroLRS model on each patient under different threshold probability. [file DataSheet_1.zip › Supplementary Figure 1.TIF]

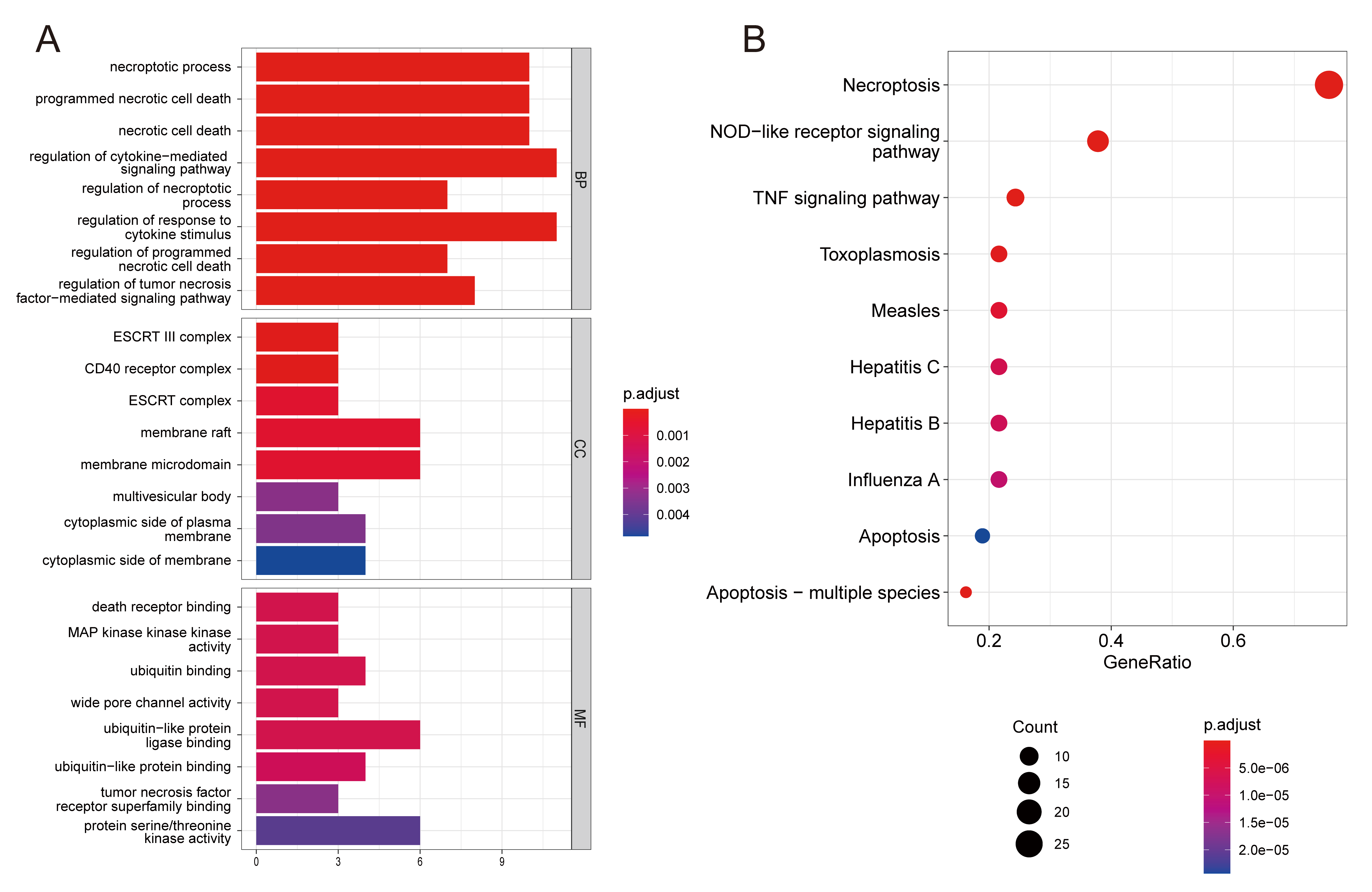

Supplement: Supplementary Figure 1 — Evaluation of NecroLRS model. Calibration curves (A–D), DCA curves (E–H) of training, test, whole, and validation cohorts (“None”: assume no patient will die at the specific time point and offer treatment to no one; “All”: assume all patients will die at the specific time point and therefore treat everyone; “Model”: gives the expected net benefit of NecroLRS model on each patient under different threshold probability. [file DataSheet_1.zip › Supplementary Figure 2.TIF]

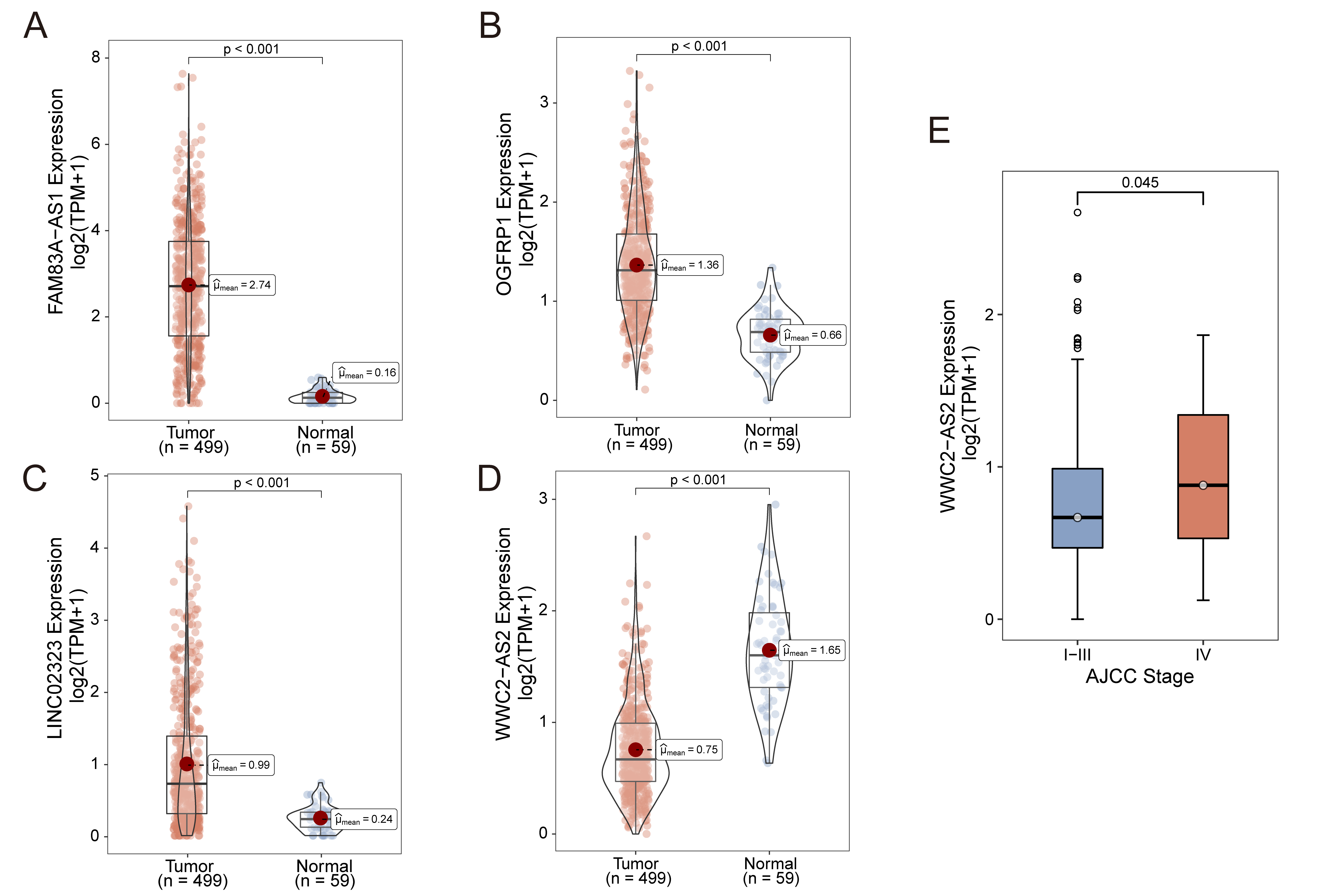

Supplement: Supplementary Figure 1 — Evaluation of NecroLRS model. Calibration curves (A–D), DCA curves (E–H) of training, test, whole, and validation cohorts (“None”: assume no patient will die at the specific time point and offer treatment to no one; “All”: assume all patients will die at the specific time point and therefore treat everyone; “Model”: gives the expected net benefit of NecroLRS model on each patient under different threshold probability. [file DataSheet_1.zip › Supplementary Figure 3.tif]

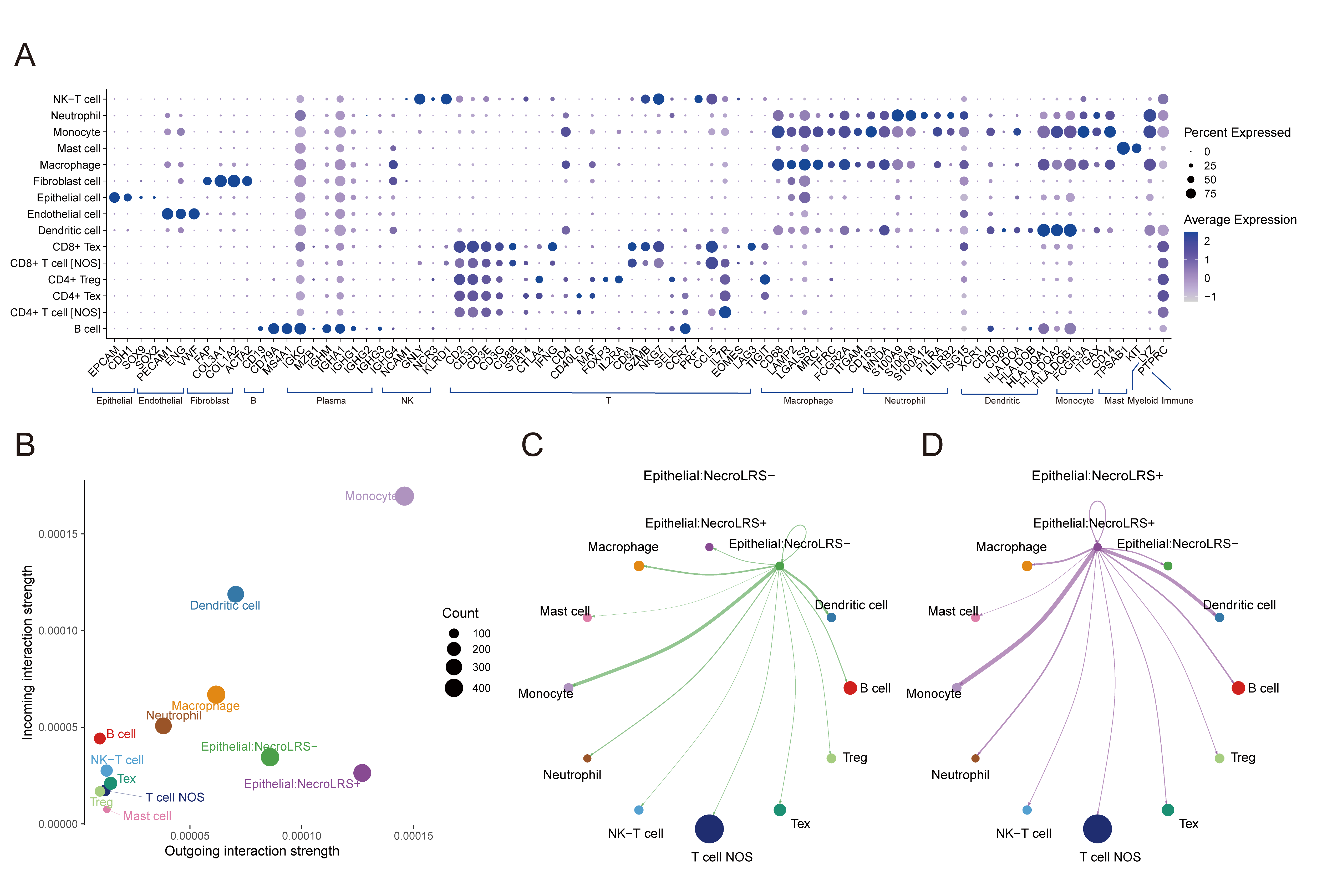

Supplement: Supplementary Figure 1 — Evaluation of NecroLRS model. Calibration curves (A–D), DCA curves (E–H) of training, test, whole, and validation cohorts (“None”: assume no patient will die at the specific time point and offer treatment to no one; “All”: assume all patients will die at the specific time point and therefore treat everyone; “Model”: gives the expected net benefit of NecroLRS model on each patient under different threshold probability. [file DataSheet_1.zip › Supplementary Figure 4.TIF]
